# Efficacy of Atezolizumab for Advanced Non-Small Cell Lung Cancer Based on Clinical and Molecular Features: A Meta-Analysis

**DOI:** 10.3389/fimmu.2022.909027

**Published:** 2022-06-21

**Authors:** Wenjie Liu, Gengwei Huo, Peng Chen

**Affiliations:** ^1^Department of Thoracic Oncology, Tianjin Medical University Cancer Institute and Hospital, National Clinical Research Center for Cancer, Key Laboratory of Cancer Prevention and Therapy of Tianjin, Tianjin’s Clinical Research Center for Cancer, Tianjin, China; ^2^Department of Oncology, Jining No. 1 People’s Hospital, Jining, China

**Keywords:** atezolizumab, non-small cell lung cancer, potency, predictor, meta-analysis

## Abstract

**Objective:**

Atezolizumab is becoming a significant therapy for non-small cell lung cancer (NSCLC), but its efficacy needs to be further improved. The aims of this study are to clarify the potency of atezolizumab-based therapy in advanced NSCLC patients with different clinical and molecular features, and to choose a better therapeutic regimen of atezolizumab to achieve more precise treatment in immunotherapy.

**Methods:**

Randomized clinical trials (RCTs) in the Cochrane Library, PubMed, Embase Science Direct, and Google Scholar, together with major oncology conferences that compared atezolizumab with chemotherapy-based treatment for individuals with advanced NSCLC published prior to February 2022, were searched. Studies, bias risk assessment, and data extraction were selected by two independent authors. We extracted the basic features of the included studies, together with the 95% confidence interval (CI) and hazard ratios (HRs), from all patients and subgroups. The combined treatment data were assessed using the inverse variance weighting method.

**Results:**

Seven RCTs including 4,859 patients were included. Our meta-analysis findings indicated that atezolizumab substantially enhanced OS (HR 0.82; 95% CI, 0.77–0.88; *p* < 0.00001) and PFS (HR 0.72; 95% CI, 0.61–0.85; *p* < 0.0001) in patients with advanced NSCLC compared with chemotherapy-based treatment. Atezolizumab substantially enhanced OS in patients aged <65 years old and 65–74 years old, those with wild-type EGFR, those without liver metastases, active or previous smokers, white patients and those with TC3 or IC3, TC2/3 or IC2/3, TC1/2/3 or IC1/2/3, and TC0 and IC0, but not in patients aged ≥75 years, never smokers, those with liver metastases, those with EGFR mutant, Asians, Black or African Americans, or those with TC1/2 or IC1/2. Patients with advanced NSCLC who received atezolizumab showed OS improvement regardless of sex (male or female), histological type (non-squamous or squamous NSCLC), performance status (0 or 1), and line of treatment (1st-line therapy or ≥2nd-line therapy). Subgroup analysis revealed that male individuals, those with non-squamous NSCLC, those with PS 1, active or previous smokers, and those with wild-type EGFR, TC3 or IC3, and TC1/2/3 or IC1/2/3 achieved OS benefit from atezolizumab treatment not related to the treatment line and treatment regimen.

**Conclusions:**

Age group, smoking history, liver metastasis status, EGFR mutation status, race, and PD-L1 expression can be used to predict the potency of atezolizumab and provide a better treatment regimen for patients with advanced NSCLC to achieve accurate and personalized treatment.

## Introduction

Lung cancer is one of the most common fatal solid malignancies worldwide and the leading cause of death (1). Non-small cell lung cancer (NSCLC) accounts for nearly 85% of all lung cancers (2). Over the past 20 years, research on immunobiology and cancer immune checkpoint blocking therapies has stimulated further interest in immunotherapy for NSCLC (3–5). Immune checkpoint inhibitors (ICIs) have become the 1st-line therapy for a variety of malignant tumors, adding immunotherapy to the ranks of surgery, chemotherapy, radiotherapy, and targeted therapy (6, 7). Atezolizumab is currently regarded as an effective treatment option for NSCLC (8), and its mechanism of action is different from other inhibitors; this kind of monoclonal antibody directly binds to programmed cell death ligand 1 (PD-L1), promoting double blockade of B7 and programmed cell death protein 1 (PD-1) receptors, thereby restoring anticancer immunity (9). The results of many large-scale randomized controlled trials (RCTs) based on atezolizumab in NSCLC patients have confirmed the concept of a durable anti-tumor response and improved overall survival (OS) and progression-free survival (PFS) (10).

However, only a few individuals (15%–25%) have observed a survival benefit, and most individuals have primary or acquired resistance to ICIs (11). Serious and life-threatening adverse events were observed in these patients. It is becoming even more urgent to explore suitable biomarkers to identify candidates for atezolizumab and achieve accurate treatment of NSCLC, both to protect individuals from ineffective treatment and to limit the number of individuals exposed to the potential autoimmune side effects of targeted axis drugs (12, 13).

To date, PD-L1 expression has emerged as the best-known and most commonly used biomarker to predict which patients are highly likely to respond to immunotherapy in NSCLC (14, 15). The association between atezolizumab and PD-L1 expression response has been explored in several NSCLC studies (16–18). Atezolizumab is more likely to benefit individuals with high levels of expression of PD-L1 reflected in tissue samples (19). Unfortunately, obtaining sufficient tumor tissue for molecular detection in individuals with advanced NSCLC is challenging. Furthermore, the lack of unification between various anti-PD-L1 clones and immunohistochemistry platforms and methodological issues, such as antibodies and positive thresholds for evaluating PD-L1 expression, are also difficult issues (13, 20, 21). Another predictive biomarker is the tumor mutation burden (TMB); high TMB has a clinical effect on atezolizumab in patients with NSCLC (22), but there is no consensus at present. Although CD8 + T cells and other emerging biomarkers have broad prospects, they also have some limitations (23–25).

It is important to identify other practical and economic factors to predict the potency of atezolizumab. There are differences in the role of atezolizumab in individuals with different clinical and molecular features (26). Therefore, we conducted this meta-analysis to determine the predictive value of various clinical and molecular attributes to guide the selection of individuals with NSCLC who would benefit from atezolizumab. The Preferred Reporting Items for Systematic Reviews and Meta-Analyses reporting checklist was used in the meta-analysis.

## Methods

### Inclusion and Exclusion Criteria

Articles that met the inclusion and exclusion criteria selected PICOs-based elements (participants, intervention, comparison, and outcomes). Prior to screening the studies by title and abstract, duplicate articles were removed from the collected studies. This was done in order to identify research papers that fulfilled the following inclusion criteria: (I) atezolizumab alone or in combination with chemotherapy ± antiangiogenic drugs compared with chemotherapy ± angiogenesis treatment for the treatment of NSCLC individuals; (II) reported hazard ratio (HR) and confidence interval (CI) 95% for OS and/or PFS with predefined subgroups, such as age group, sex, histological type, Eastern Cooperative Oncology Group (ECOG) performance status (PS) score, smoking status, liver metastasis status, EGFR mutation status, race, the expression of PD-L1 in tumor-infiltrating immune cells (IC) or tumor cells (TC), and treatment line; (III) multiple studies confirmed the same trial, using the latest data with the longest follow-up and the largest patient population; (IV) we incorporated all of the distinct subgroups if multiple studies were described from the same clinical trial.

The following criteria were applied to exclude the studies involved: (I) does not distinguish between the effects of multiple ICIs, and (II) inadequate existing survival data, or the control group garnered only a placebo. For information resources, we refer not only to the full text of the article but also to the appendix and the references listed at the end of each article.

### Literature Search and Data Collection

The selection of research and extraction of data were independently completed by two authors (WL and GH). If any ambiguity was encountered, a third author was sought (PC). We searched a variety of electronic databases, including Cochrane Library, PubMed, Embase Science Direct, and Google Scholar, together with major oncology conferences. The main browse terms were randomized clinical trials, atezolizumab, NSCLC, potency, efficacy, and predictor, and other words were also added. The articles were published prior to February 2022 according to the search criteria. The information for each study was recorded as follows: trial name, publication year, first author, study phase, treatment line, age composition, sex composition, smoking status, histological type, PD-L1 expression, ECOG score, primary endpoint, clinical trial design and blinding, as well as the survival outcome measures of predefined subgroups.

### Quality Assessment and Statistical Analyses

The validity and reliability of the study were evaluated by two researchers who worked independently (WL and GH) using the Cochrane bias tool. All statistical analyses were performed using the statistical software Review Manager version 5.3. The main endpoint of the study was to compare OS between atezolizumab-based therapy and chemotherapy-based treatment, which was measured by HR and the corresponding CI. PFS was used as a secondary endpoint. HR was calculated using either fixed-effects models or random effects based on the heterogeneity of the studies included in the analysis. The existence of heterogeneity was tested using the *I*^2^ statistic test and chi-square test. A fixed-effects model was used when heterogeneity was considered acceptable (*I*^2^ <50% and *p* > 0.10); otherwise, the random-effects model was used. Because the treatment of interest is typically evaluated in a single trial, fixed-effects models are employed. The results are presented as forest plots, along with pooled summary estimates and 95% CI that correspond to these estimates. The logarithmic scales on forest plots were used to manually extract HR and 95% CI when they were not reported directly by the authors in the text.

Sensitivity analysis was conducted by excluding studies for which the HR and associated 95% CI could not be obtained directly from the studies themselves or with a small sample size. The nominal level of significance was set at *p* < 0.05.

## Results

### Study Selection and Characteristics

**Figure 1:**
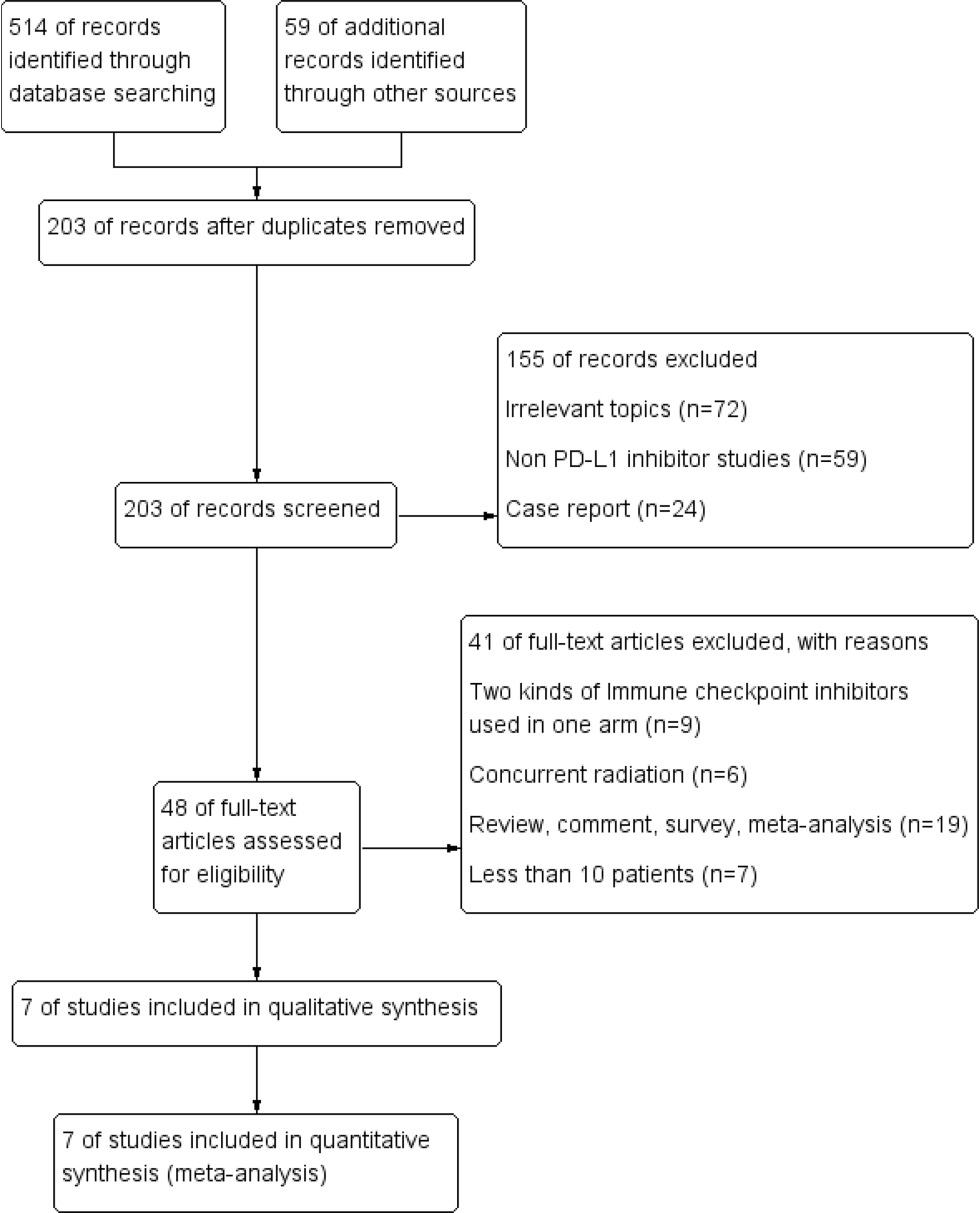
PRISMA flow diagram.

**Figure 2:**
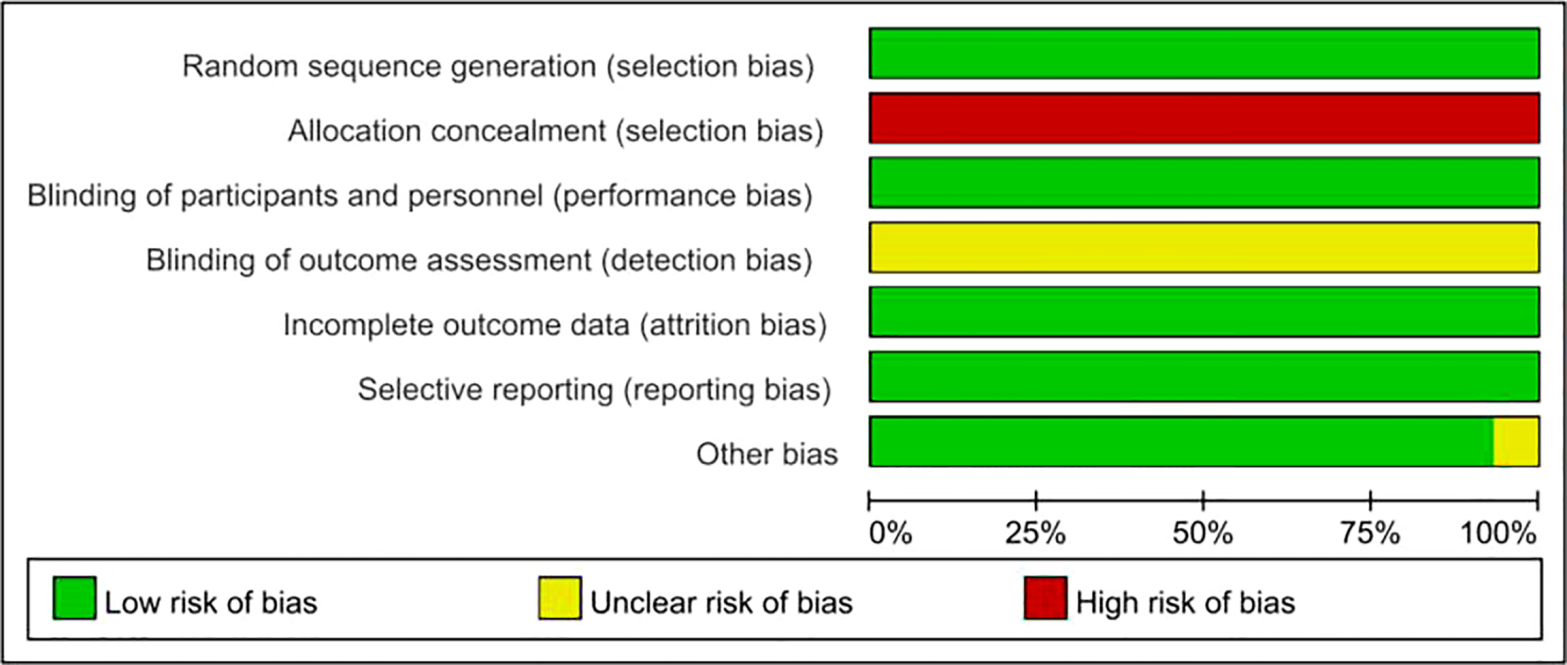
Risk of bias graph.

A total of 573 potentially relevant records were identified from the databases and conferences as a result of the search strategy employed in the research. **Figure 1** depicts the selection process, as well as the rationale for excluding studies deemed ineligible. A total of 566 studies were excluded after screening for their abstracts and full texts. Thus, 7 RCTs involving 4,859 individuals with advanced NSCLC were included in the meta-analysis (**Table 1**). These RCTs were published between 2016 and 2021 and were divided into the following categories: one of the studies was a clinical trial in phase II (18), and six were phase III trials (19, 27–36). Detailed risk of bias analysis revealed that the risk of bias in all RCTs was low (**Figures 2**, **S1**).

**Table 1:** Basic characteristics of included studies.

Reference	Trial	Study phase	Stage	Treatment line	ICI used (n)	Control arm (n)	Histological type		Median(range) Age (years)	Male (%)	Never smokers (%)	Tumor PD-L1 expression			ECOG		Primary endpoint
							Squamous (%)	Non-squamous (%)				<1% (%)	≥1% (%)	Unknown (%)	0(%)	1 (%)	
Fehrenbacher (18)	POPLAR	II	Advanced or metastatic	≥2L	Atezolizumab (144)	Docetaxel (143)	33.8	66.2	62.0 (36–84)	58.9	19.5	62	38	0	31.7	67.2	OS
Rittmeyer (27)	OAK	III	IIIB or IV	≥2L	Atezolizumab (613)	Docetaxel (612)	26.2	73.8	63.5 (25–85)	61.9	17	43.3	55.8	0.8	37.1	62.9	OS
Fehrenbacher (28)																	
Socinski (29)	IMpower 150	III	IV or recurrent metastatic	1L	Atezolizumab + carboplatin + paclitaxel (402)	Bevacizumab + carboplatin + paclitaxel (400)	0	100	63.0 (31–90)	60	19.9	48.8	50.5	0.7	42.2	57	PFS and OS
Reck (30)																	
Socinski (31)					Atezolizumab + bevacizumab + carboplatin + paclitaxel (400)												
Nogami (32)																	
West (33)	IMpower 130	III	IV	1L	Atezolizumab + carboplatin + nab-paclitaxel (451)	Carboplatin + nab-paclitaxel (228)	0	100	64.3 (18–86)	58.9	9.6	52.4	47.6	0	41.2	58.5	PFS and OS
Jotte (34)	IMpower 131	III	IV	1L	Atezolizumab + carboplatin + nab-paclitaxel (343)	Carboplatin + nab-paclitaxel (340)	100	0	65.0 (23–86)	81.6	8	48.5	51.4	0.1	32.9	66.8	PFS and OS
Nishio (35)	IMpower 132	III	IV	1L	Atezolizumab (292)	Carboplatin or cisplatin plus pemetrexed (286)	0	100	63.5 (31-85)	66.4	11.6	28.2	31.3	40.5	41.5	58.1	PFS and OS
Herbst (19)	IMpower 110	III	IV	1L	Atezolizumab (107)	Chemotherapy (98)	24.4	75.6	64.4 (33-87)	69.8	11.7	0	100	0	35.6	64.4	OS
Jassem (36)																	

### Effects of Atezolizumab in NSCLC

**Figure 3:**
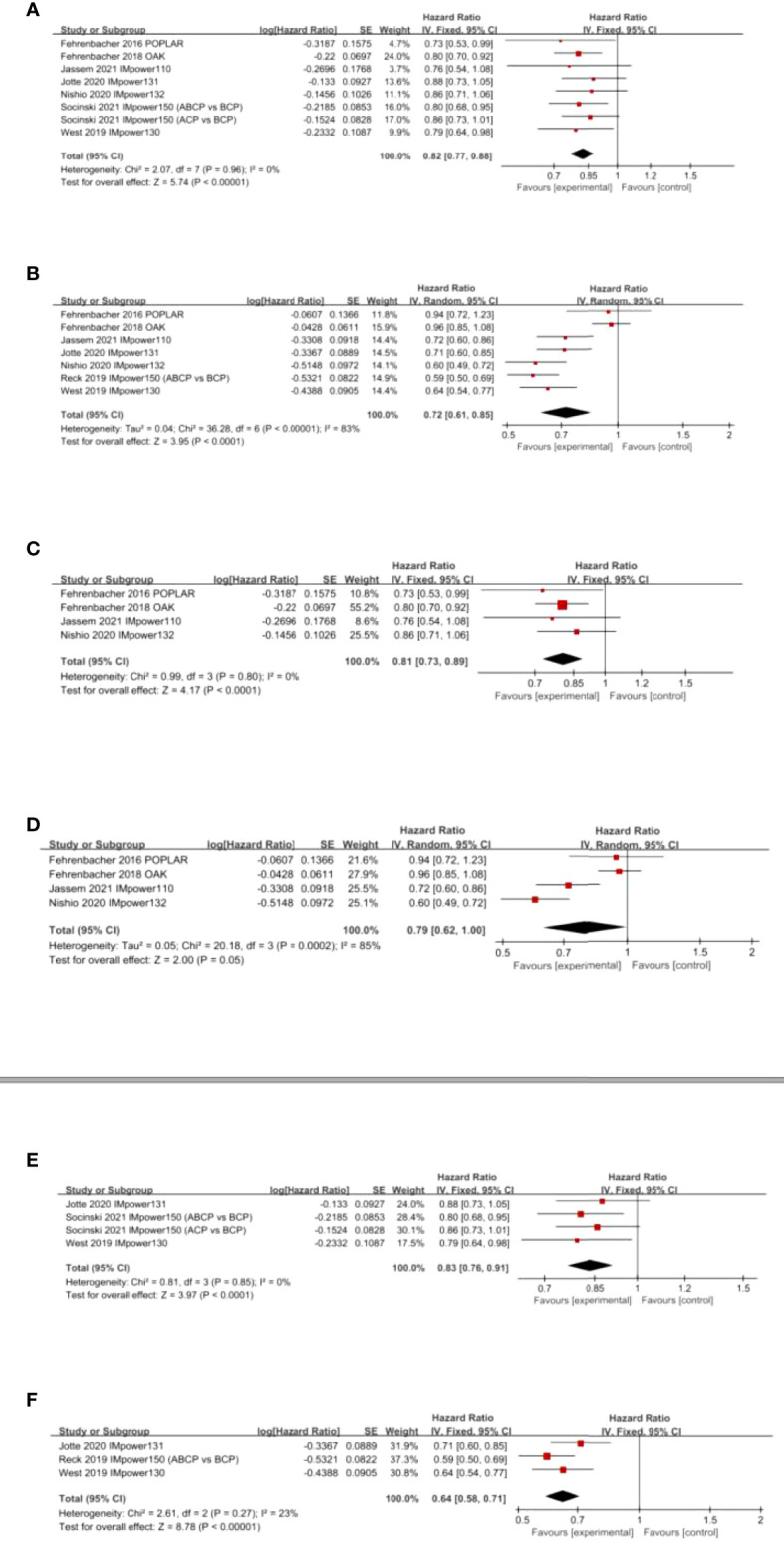
Forest plots of HRs comparing **(A)** OS and **(B)** PFS between atezolizumab-based therapy and chemotherapy-based therapy, **(C)** OS and **(D)** PFS based on atezolizumab monotherapy, and **(E)** OS and **(F)** PFS based on atezolizumab combined therapy.

Eight studies examined the efficacy of atezolizumab-based therapy in NSCLC compared with chemotherapy-based treatment. The comprehensive results showed that atezolizumab substantially enhanced OS (HR 0.82; 95% CI, 0.77–0.88; *p* < 0.00001) (**Figure 3A**). Seven investigations reported that atezolizumab-based therapy achieved PFS improvement in NSCLC patients compared to chemotherapy-based treatment (HR 0.72; 95% CI, 0.61–0.85; *p* < 0.0001) (**Figure 3B**). Based on the treatment regimens, atezolizumab combined with chemotherapy significantly prolonged patients’ PFS compared to chemotherapy alone; however, this survival benefit was not observed with atezolizumab monotherapy. In addition, a significant improvement in OS was observed with atezolizumab monotherapy or combination therapy (**Figures 3C–F**).

### Effects of Atezolizumab by Age Group

**Figure 4:**
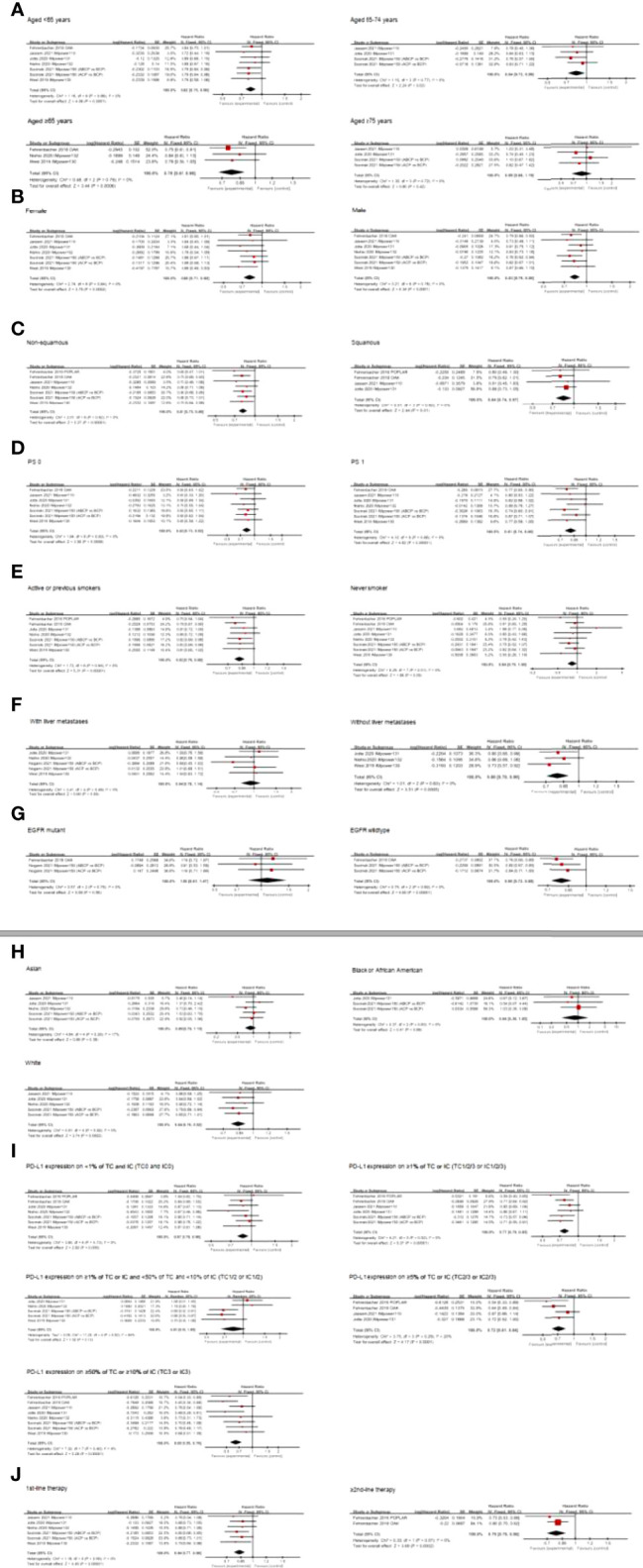
Forest plots of HRs comparing OS between atezolizumab-based therapy and chemotherapy-based therapy with respect to **(A)** age group, **(B)** gender, **(C)** histological type, **(D)** PS score, **(E)** smoking status, **(F)** liver metastases status, **(G)** EGFR mutation status, **(H)** race, **(I)** PD-L1expression, and **(J)** treatment line.

Age group-specific survival data for individuals with NSCLC were presented in seven articles. In individuals aged <65 years (HR 0.82; 95% CI, 0.75–0.90; *p* < 0.0001) and those aged ≥65 years (HR 0.78; 95% CI, 0.67–0.90; *p* = 0.0006), atezolizumab-based therapy substantially increased OS relative to chemotherapy-based treatment. Interestingly, when the cutoff value of age was set at 65–74 years old and ≥75 years old, we discovered an OS benefit in individuals aged 65–74 years old (HR 0.84; 95% CI, 0.72–0.98; *p* = 0.02), while no OS benefit was observed in patients aged ≥75 years (HR 0.89; 95% CI, 0.66-1.19; *p* = 0.42) (**Figure 4A**).

Subgroup analyses based on the treatment regimen showed that this factor did not affect the OS improvement with atezolizumab in individuals aged <65 years. Subgroup analyses based on the line of therapy showed that the combined HR of six studies in 1st-line therapy was 0.82 (95% CI, 0.73–0.91; *p* = 0.0003). Only one study was related to ≥2nd-line therapy, with an HR of 0.84 (95% CI, 0.70–1.01; *p* = 0.06). In addition, atezolizumab significantly improved OS in individuals aged 65–74 years old who received 1st-line combination therapy (HR 0.84; 95% CI, 0.72–0.99; *p* = 0.04), but not those in 1st-line monotherapy. However, no prolonged survival was observed in individuals aged ≥75 years regardless of 1st-line monotherapy and 1st-line combination therapy. (**Table S1**). For PFS data from four studies, atezolizumab-based therapy substantially enhanced survival compared with chemotherapy-based treatment in patients aged <65 years (HR 0.67; 95% CI, 0.59–0.76; *p* < 0.00001) and ≥65 years (HR 0.60; 95% CI, 0.50–0.72; *p* < 0.00001). Similarly, we observed PFS benefit in individuals aged 65–74 years old (HR 0.60; 95% CI, 0.49–0.73; *p* < 0.00001) and ≥75 years old (HR 0.62; 95% CI, 0.43–0.90; *p* = 0.01) (**Figure S2A** and **Table S2**).

### Effects of Atezolizumab by Gender

Seven studies examined the potency of atezolizumab-based therapy in both female and male individuals with respect to OS. The comprehensive results showed that atezolizumab substantially enhanced OS in both sexes of NSCLC patients compared with chemotherapy-based treatment (HR 0.80; 95% CI, 0.71–0.90; *p* = 0.0002 for female patients; HR 0.83; 95% CI, 0.76–0.90; *p* < 0.0001 for male patients) (**Figure 4B**). Subgroup analyses showed that in male patients, atezolizumab substantially enhanced OS, which was not related to treatment line and treatment regimen. In female patients, we found that atezolizumab improved OS in 1st-line therapy (HR 0.80; 95% CI, 0.70–0.91; *p* = 0.0010), but not in ≥2nd-line therapy. Atezolizumab enhanced OS in both monotherapy (HR 0.80; 95% CI, 0.67–0.96; *p* = 0.01) and combination therapy (HR 0.80; 95% CI, 0.69–0.93; *p* = 0.004) (**Table S1**). PFS data from four studies showed substantially enhanced PFS in female patients (HR 0.62; 95% CI, 0.53–0.73; *p* < 0.00001) and in male patients (HR 0.64; 95% CI, 0.58–0.72; *p* < 0.00001) (**Figure S2B** and **Table S2**).

### Effects of Atezolizumab by Histological Type

The potency of atezolizumab-based therapy for non-squamous and squamous NSCLC was studied in seven and four studies, respectively. The integrated findings revealed that atezolizumab enhanced OS in both non-squamous NSCLC (HR 0.81; 95% CI, 0.75–0.88; *p* < 0.00001) and squamous cell carcinoma (HR 0.84; 95% CI, 0.74–0.97; *p* = 0.01) (**Figure 4C**). Subgroup analyses showed that in non-squamous NSCLC individuals, atezolizumab substantially enhanced OS, which was not related to treatment line or treatment regimen. In squamous NSCLC patients, atezolizumab only benefited from ≥2nd-line treatment (HR 0.79; 95% CI, 0.64–0.99; *p* = 0.04) and monotherapy (HR 0.80; 95% CI, 0.65–0.99; *p* = 0.04) (**Table S1**). PFS data from five studies showed substantially enhanced PFS in non-squamous NSCLC individuals (HR 0.61; 95% CI, 0.55–0.67; *p* < 0.00001), but not in squamous NSCLC individuals (HR 0.80; 95% CI, 0.62–1.05; *p* = 0.11) (**Figure S2C** and **Table S2**).

### Effects of Atezolizumab by ECOG PS Score

Seven studies examined the effectiveness of atezolizumab-based therapy in individuals with PS scores of 0 and 1. The integrated results revealed that compared with chemotherapy-based therapy, individuals with PS 0 (HR 0.82; 95% CI, 0.73–0.92; *p* = 0.0008) and PS 1 (HR 0.81; 95% CI, 0.74–0.88; *p* < 0.00001) realized OS enhancements after applying atezolizumab (**Figure 4D**). For patients with PS 0, subgroup analyses by the treatment line showed that atezolizumab only benefits from 1st-line treatment (HR 0.83; 95% CI, 0.72–0.94; *p* = 0.004), but did not benefit from ≥2nd-line therapy. Based on the treatment regimen, subgroup analyses showed that atezolizumab enhanced OS in both monotherapy (HR 0.77; 95% CI, 0.64–0.93; *p* = 0.006) and combination therapy (HR 0.85; 95% CI, 0.74–0.99; *p* = 0.04). In patients with PS 1, atezolizumab substantially enhanced OS unrelated to the treatment line and treatment regimen (**Table S1**). PFS data from four studies showed substantially enhanced PFS both in individuals with PS 0 (HR 0.59; 95% CI, 0.51–0.69; *p* < 0.00001) and in individuals with PS 1 (HR 0.66; 95% CI, 0.59–0.74; *p* < 0.00001) (**Figure S2D** and **Table S2**).

### Effects of Atezolizumab by Smoking Status

Atezolizumab-based therapy was found to be more effective than chemotherapy-based treatment in improving OS in individuals who either were actively smoking or had previously smoked (HR 0.82; 95% CI, 0.76–0.88; *p* < 0.00001) (**Figure 4E**). Subgroup analyses showed that in active or previous smokers, atezolizumab substantially enhanced OS unrelated to treatment line and treatment regimen (**Table S1**). Individuals who had never smoked who received atezolizumab and those who received conventional treatment had no statistically significant difference in OS (HR 0.84; 95% CI, 0.70–1.00; *p* = 0.05) (**Figure 4E**). Analysis of subgroups revealed that no prolonged OS was observed in never-smokers, and in either therapy line or treatment regimen (**Table S1**). PFS data from four studies showed substantially enhanced PFS in individuals who were active or former smokers (HR 0.63; 95% CI, 0.58–0.70; *p* < 0.00001) and in individuals who never smoked (HR 0.68; 95% CI, 0.52–0.89; *p* = 0.004) (**Figure S2E** and **Table S2**).

### Effects of Atezolizumab by Liver Metastatic Status

In individuals with liver metastases, five studies reported data on OS and PFS. Individuals on atezolizumab-based therapy had an OS rate of 0.94, with a 95% CI of 0.78–1.14 (*p* = 0.55), and a prolonged PFS rate of 0.71, with a 95% CI of 0.54–0.94 (*p* = 0.02) (**Figures 4F** and **S1F**). Subgroup analyses showed that in individuals with liver metastases, there was no significant difference in OS between the two groups, either on the treatment line or on the treatment regimen (**Tables S1**, **S2**). Individuals without liver metastases were the focus of three studies that examined OS. Individuals without liver metastases who received atezolizumab-based therapy had a longer OS (HR 0.80; 95% CI, 0.70–0.90; *p* = 0.0005) than those who received chemotherapy-based treatment (**Figure 4F**). Subgroup analyses showed that in individuals without liver metastases, atezolizumab benefits from 1st-line treatment (HR 0.80; 95% CI, 0.70–0.90; *p* = 0.0005) and combination therapy (HR 0.77; 95% CI, 0.66–0.90; *p* = 0.0010) (**Table S1**). In terms of PFS, we also observed survival benefits in patients without liver metastases (HR 0.62; 95% CI, 0.56–0.68; *p* < 0.00001) (**Figure S2F** and **Table S2**).

### Effects of Atezolizumab by EGFR Mutation Status

Results in terms of OS were published in three studies, and combined results showed that atezolizumab provided longer OS for EGFR wild-type individuals (HR 0.80; 95% CI, 0.72–0.88; *p* < 0.00001), but not for EGFR mutant individuals (HR 1.09; 95% CI, 0.81–1.47; *p* = 0.56) compared with chemotherapy-based treatment (**Figure 4G**). The results were not affected by the treatment line or regimen in our subgroup analyses. In terms of PFS, we similarly did not observe a PFS benefit in EGFR mutation-positive individuals (HR 0.85; 95% CI, 0.46–1.56; *p* = 0.60), but a survival benefit in EGFR wild-type individuals (HR 0.62; 95% CI, 0.52–0.74; *p* < 0.00001) was observed (**Figure S2G** and **Table S2**).

### Effects of Atezolizumab by Race

For white patients, the effectiveness of atezolizumab-based therapy has been demonstrated in five clinical trials. OS was substantially improved by atezolizumab compared to chemotherapy-based treatment (HR 0.84; 95% CI, 0.76–0.92; *p* = 0.0002), according to the combined data (**Figure 4H**). Subgroup analyses showed that atezolizumab substantially enhanced OS in 1st-line combination therapy (HR 0.82; 95% CI, 0.74–0.91; *p* = 0.0003), but not in 1st-line monotherapy (**Table S1**). The potency of atezolizumab-based therapy for Asian and Black or African American NSCLC patients was studied in five and three studies, respectively. The integrated findings revealed that atezolizumab-based therapy did not obviously enhance OS in both Asian (HR 0.89; 95% CI, 0.70–1.15; *p* = 0.38) and Black or African American patients with NSCLC (HR 0.84; 95% CI, 0.36–1.95; *p* = 0.68) (**Figure 4H**). In terms of PFS, atezolizumab-based therapy improved PFS in Asian (HR 0.53; 95% CI, 0.32–0.86; *p* = 0.010) and White (HR 0.69; 95% CI, 0.60–0.79; *p* < 0.00001) patients compared to chemotherapy-based treatment, but did not prolong survival in Black or African American patients with NSCLC (HR 0.39; 95% CI, 0.07–2.08; *p* = 0.27) (**Figure S2H** and **Table S2**).

### Effects of Atezolizumab by PD-L1 Expression

Seven studies examined the potency of atezolizumab-based therapy in individuals with PD-L1 expression on <1% of TC and IC (TC0 and IC0) and showed that atezolizumab substantially enhanced OS when compared to chemotherapy-based treatment (HR 0.87; 95% CI, 0.78–0.96; *p* = 0.005) (**Figure 4I**). Subgroup analyses showed that atezolizumab benefits from 1st-line treatment (HR 0.86; 95% CI, 0.77–0.97; *p* = 0.02) and monotherapy (HR 0.82; 95% CI, 0.69–0.97; *p* = 0.02) (**Table S1**).

Six studies reported the potency of atezolizumab in individuals with PD-L1 expression on ≥1% of TC or IC (TC1/2/3 or IC1/2/3). The aggregated findings indicated that atezolizumab prolonged the OS (HR 0.77; 95% CI, 0.70–0.85; *p* < 0.00001) (**Figure 4I**). Subgroup analyses showed that atezolizumab substantially enhanced OS unrelated to the treatment line and treatment regimen (**Table S1**).

In three trials that examined the effectiveness of atezolizumab-based therapy in individuals with PD-L1 expression on ≥1% TC or IC and <50% TC and <10% IC (TC1/2 or IC1/2), there was no significant difference in OS between atezolizumab and chemotherapy (HR 0.83; 95% CI, 0.65–1.06; *p* = 0.13) (**Figure 4I**). Analysis of subgroups showed that prolonged survival was observed in 1st-line combination therapy based on atezolizumab (HR 0.77; 95% CI, 0.60–0.98; *p* = 0.04), but not in monotherapy (**Table S1**).

In four studies that examined the potency of atezolizumab in individuals with PD-L1 expression on ≥5% of TC or IC (TC2/3 or IC2/3), it was discovered that atezolizumab substantially enhanced OS when compared to chemotherapy-based therapy (HR 0.72; 95% CI, 0.61–0.84; *p* < 0.0001) (**Figure 4I**). Analysis of subgroups by the treatment line showed that receiving 1st-line treatment (HR 0.80; 95% CI, 0.65–0.99; *p* = 0.04) and ≥2nd-line treatment (HR 0.62; 95% CI, 0.49–0.78; *p* < 0.0001) both prolonged the patient’s OS. Atezolizumab improved OS as monotherapy (HR 0.71; 95% CI, 0.60–0.85; *p* = 0.0002), but not as a combination therapy (**Table S1**).

According to the cumulative findings from eight studies, atezolizumab-based therapy markedly enhanced OS over chemotherapy-based treatment in individuals with PD-L1 expression on ≥50% TC or ≥10% IC (TC3 or IC3) (HR 0.65; 95% CI, 0.55–0.76; *p* < 0.00001) (**Figure 4I**). Atezolizumab substantially enhanced OS unrelated to the treatment line and treatment regimen according to subgroup analyses (**Table S1**).

Seven studies reported PFS of individuals with NSCLC stratified by PD-L1 expression, and we found that only individuals with PD-L1-negative expression did not observe a PFS benefit (HR 0.80; 95% CI, 0.63–1.01; *p* = 0.07), whereas all other individuals with PD-L1-positive expression achieved PFS benefits in atezolizumab-based therapy (**Figure S2I** and **S2**).

### Drug Selection

Clinical and molecular characteristics could be used to predict the efficacy of atezolizumab in different treatment lines and regimens, as shown in **Tables 2** and **S3**. According to our pooled results, atezolizumab-based therapy had significantly enhanced OS over chemotherapy-based treatment in 1st-line and ≥2nd-line treatment in individuals with specific features (HR 0.84; 95% CI, 0.77–0.90; *p* < 0.00001 for 1st-line therapy; HR 0.79; 95% CI, 0.70–0.90; *p* = 0.0002 for ≥2nd-line therapy) (**Figure 4J**). Analysis of subgroups showed that in 1st-line treatment, atezolizumab combination therapy substantially enhanced OS compared to chemotherapy-based treatment in patients aged <65 years old; those aged 65–74 years old; male patients and female patients; patients with non-squamous NSCLC, PS 0, PS 1, and EGFR wild-type; those without liver metastasis; active or previous smokers; white patients; and those with TC1/2/3 or IC1/2/3, TC1/2 or IC1/2, and TC3 or IC3. In ≥2nd-line treatment, atezolizumab monotherapy substantially prolonged the OS of patients aged ≥65 years; male patients; those with squamous NSCLC, non-squamous NSCLC, and PS 1; active or previous smokers; and those with EGFR wild-type, TC1/2/3 or IC1/2/3, TC2/3 or IC2/3, and TC3 or IC3. In addition, TC0 and IC0, as well as PS 0 showed survival benefits in 1st-line monotherapy. In terms of PFS, we observed a survival benefit in patients receiving 1st-line based on atezolizumab (HR, 0.65; 95%CI, 0.60–0.70; *p* < 0.00001), but not in those receiving ≥2nd-line treatment (HR, 0.96; 95%CI, 0.86–1.07; *p* = 0.41) (**Figure S2J** and **Table S5**).

**Table 2:** Different treatment lines and regimens with OS benefited from atezolizumab-based therapy over chemotherapy-based therapy in targeted patients.

Line	Regimen	Population	No. of studies	HR	95% CI	*p*-value
1st-Line	Combined therapy	Aged <65 years	4	0.81	0.72–0.92	0.0007
		Aged 65–74 years	3	0.84	0.72–0.99	0.04
		Male	4	0.84	0.75–0.93	0.001
		Female	4	0.80	0.69–0.93	0.004
		Non-squamous	3	0.82	0.74–0.91	0.0002
		PS 0	4	0.85	0.74–0.99	0.04
		PS 1	4	0.80	0.72–0.90	<0.0001
		Active or previous smoker	4	0.83	0.76–0.92	0.0002
		Without liver metastases	2	0.77	0.66–0.90	0.0010
		EGFR wildtype	2	0.82	0.73–0.93	0.002
		White	3	0.82	0.74–0.91	0.0003
		TC1/2/3 or IC1/2/3	3	0.76	0.66–0.88	0.0003
		TC1/2 or IC1/2	4	0.77	0.60–0.98	0.04
		TC3 or IC3	4	0.69	0.55–0.87	0.002
	Monotherapy	PS 0	2	0.73	0.55–0.97	0.03
		TC0 and IC0	1	0.67	0.46–0.96	0.03
≥2nd-Line	Monotherapy	Aged ≥65 years	1	0.75	0.61–0.91	0.004
		Male	1	0.79	0.66–0.93	0.005
		Squamous	2	0.79	0.64–0.99	0.04
		Non-squamous	2	0.78	0.67–0.90	0.0008
		PS 1	1	0.77	0.65–0.90	0.001
		Active or previous smoker	2	0.77	0.67–0.88	0.0002
		EGFR wildtype	1	0.76	0.65–0.89	0.0006
		TC1/2/3 or IC1/2/3	2	0.73	0.62–0.86	0.0002
		TC2/3 or IC2/3	2	0.62	0.49–0.78	<0.0001
		TC3 or IC3	2	0.49	0.35–0.67	<0.00001

### Sensitivity Analysis and Publication Bias

The two trials of POPLAR and IMpower110 included a small number of individuals; thus, sensitivity analysis was conducted by excluding these two trials. The findings showed that a large number of clinical and molecular therapies based on atezolizumab remained stable in predicting OS during the analysis. In addition, when the Socinski 2018 study, included in the IMpower150 trial, was excluded, which only provided HR, the 95% CI was estimated from the forest plot, and we discovered that the preliminary analysis conclusion did not change. In addition, we found no significant publication bias according to overall OS and PFS funnel of the whole (**Figure S3**) and subgroups (**Figures S4** and **S5**).

## Discussion

We used the most recent clinical data and sought to determine whether practical and economical clinical and molecular pathological markers are available, which can be used to predict the potency of atezolizumab and guide treatment options for populations that may benefit from atezolizumab in the field.

Preclinical and clinical data suggest that whether PD-L1 inhibitors are beneficial to elderly patients with NSCLC remains controversial (37–40). It is still unclear whether PD-L1 inhibitors, such as atezolizumab, will be used in elderly patients. In individuals aged ≥75 years, our results did not show that atezolizumab was more effective than chemotherapy-based treatment for OS. This may be due to the decline in immune system function in the elderly, which makes them unable to restore anti-tumor activity (39), and it may also be due to the vulnerability of elderly patients to more severe immune-related toxicity than young patients (41). Thus, caution should be exercised when using atezolizumab in patients aged ≥75 years. These results were similar to those of a meta-analysis by Elias et al., which showed that PD-1/PD-L1 inhibitors provided benefits in solid tumor individuals aged <65 and ≥65 years, but not in patients aged ≥75 years (42). It is noted that according to our results, early atezolizumab-based combination therapy is recommended for individuals aged <65 years. In individuals aged 65–74 years old, the potency of ≥2nd-line treatment based on atezolizumab needs to be further clarified.

Gender variables are known to affect both innate and adaptive immune responses (43). The effect of individual sex on the potency of atezolizumab in the treatment of NSCLC remains controversial (44–48). Our meta-analysis showed that OS and PFS were both improved in male and female individuals administered atezolizumab compared with those administered chemotherapy-based treatment. However, in women, we did not find a survival benefit in the ≥2nd-line based on atezolizumab. This may be the reason for the small size of these populations. Furthermore, 1st-line treatment is more effective in female patients, probably because of a more effective immune system and limited disease burden (19). Therefore, it is worth noting that in future studies, there is more concern about the potency of female patients in ≥2nd-line treatment based on atezolizumab.

The RCTs of IMpower130 and 131 have similar clinical characteristics and the same treatment regimens, with the exception of different histological types, while different OS results were observed; IMpower130 (non-squamous) achieved an OS benefit, but IMpower131 (squamous) did not. This difference in results intrigued us and indicated that histological type may be a non-negligible factor affecting the potency of atezolizumab. Our meta-analysis results showed that for individuals with non-squamous cell NSCLC, both OS and PFS were improved regardless of the treatment line or regimen. However, squamous cell NSCLC only benefits the ≥2nd-line and monotherapy based on atezolizumab in OS, but not in first-line or combination therapy. In terms of PFS, we did not observe enhanced survival in patients with squamous cell NSCLC. The reason may be that squamous NSCLC accounts for 20%–30% of all lung cancer tissue types, whose characteristics are different from those of non-squamous NSCLC, and its prognosis is more serious (49). In addition, individuals with squamous NSCLC are older, have a higher comorbidity burden, have a history of smoking exposure, and may have clinical characteristics of nephrotoxicity (8, 50); therefore, treatment is not as effective as non-squamous cell carcinoma. Additionally, we found that atezolizumab, as an effective treatment option for NSCLC, is not histologically restricted to ≥2nd-line treatment. Based on the results of our analysis, histological type does not seem to be an appropriate predictor for evaluating the potency of atezolizumab.

The introduction of ICIs significantly improved the prognosis of NSCLC patients but was limited to ECOG PS 0 or 1 (51). In our meta-analysis, both PS 0 and 1 patients administered atezolizumab achieved OS and PFS benefits compared with those administered chemotherapy-based treatment. Therefore, an ECOG PS of 0 or 1 does not seem to be an appropriate predictor for evaluating the potency of atezolizumab. Because most RCTs excluded individuals with poorer PS (PS ≥2), we could not study the effect of atezolizumab on the PS ≥2 population. A meta-analysis of 19 real-world clinical studies revealed that a poorer survival rate existed in individuals receiving immunotherapy with PS≥2 (52). If PS ≥2 predicts poor atezolizumab potency, it should be verified by RCTs in the future.

The potency of atezolizumab in various smoking statuses was also analyzed in our analysis, and it was found that the survival benefit of atezolizumab was observed in active or former smokers but not in those who had never smoked. Some studies have shown that in NSCLC, smokers have a favorable trend for ICIs compared with non-smokers (53–55), which is consistent with our results. This may be because smoking is thought to increase the mutation load in tumors, increasing the expression of carcinogenic neo-antigen, and thus activating an efficient anti-tumor immune response (56, 57). Preclinical and clinical studies have shown that higher mutation and neoantigen loads are related to long-lasting clinical benefits of immunotherapy (58–60). This may explain why molecular smoking characteristics are associated with atezolizumab potency in NSCLC. Therefore, smoking history is a powerful clinical biomarker for the choice of atezolizumab therapy.

Due to a particularly unfavorable prognosis in NSCLC patients with distant metastases (e.g., liver metastasis) (61–63), immunotherapy has become an important treatment option for these individuals at present. Our meta-analysis showed that PFS, but not OS, was a statistically significant benefit in individuals with liver metastases treated with atezolizumab. Notably, we further found that the improvement in PFS was mainly reflected in atezolizumab 1st-line combination therapy for liver metastases. For patients without liver metastasis, OS was improved, which is similar to the atezolizumab 1st-line combined chemotherapy, but not monotherapy. These results suggest that NSCLC patients with and without liver metastasis may benefit more from combination atezolizumab treatment. A previous study showed that PD-1/PD-L1 inhibitors plus chemotherapy can lower the risk of progression by 29% and the risk of death by 21% in patients with liver metastases (64). Shiroyama et al. found that NSCLC patients with liver metastases were significantly younger and had more metastatic sites and poorer baseline ECOG PS, and lung cancer patients with these characteristics also have poorer prognoses (65). Moreover, liver metastases usually demonstrate incomplete activation of CD8 + T cells (66), deficient activation of CD8 + T cells (67), CD4 + T-cell inactivation (68), and Kupffer cells-induced regulatory T-cell activation (16), which reduced the probability of atezolizumab responding to liver metastases and did not improve OS. Consequently, liver metastatic status may be a survival outcome-independent predictor in NSCLC individuals given with atezolizumab. In addition, the strategy of atezolizumab combination therapy can expand the range of patients who respond to immunotherapy and improve the quality of clinical response, which is more than the effect of monotherapy. In addition, there are no data based on atezolizumab as a 2nd-line treatment, which deserves further exploration.

The relationship between atezolizumab and driver mutations has long been a hot research topic. In this study, we discovered that EGFR mutation status was linked to the potency of atezolizumab-based therapy. Individuals with wild-type EGFR benefited from atezolizumab treatment, whereas those with EGFR mutations did not. The biological rationale behind the effect of EGFR mutation status on the benefit of atezolizumab may be related to the fact that EGFR-mutant NSCLC subpopulations have more active escape/resistance pathways, which may limit the efficacy of atezolizumab in this context (69). Additionally, in EGFR mutant NSCLC, it is possible that atezolizumab is ineffective due to the fact that, first, previous studies indicated that the expression of PD-L1 is substantially lower in EGFR mutant tumors than in EGFR wild-type tumors, resulting in defective response to atezolizumab therapy in EGFR mutant patients (70, 71). Second, individuals with EGFR-sensitive mutations were more common in non-smokers, and their TMB was substantially lower than those with wild-type EGFR, suggesting that TMB may be a contributing factor to the poor potency of atezolizumab in EGFR-mutant patients (72–75). Third, unlike wild-type EGFR patients, EGFR mutations affect anti-tumor immune responses by modulating factors that may be associated with tumor microenvironmental status (for instance, tumor-infiltrating lymphocytes, regulatory T cells, and exosome CD73) (76–78). Although individuals with EGFR mutations usually do not respond well to atezolizumab, some can benefit from immunotherapy. OS was improved in EGFR mutation-sensitive individuals (19DEL and L858R) after atezolizumab plus chemotherapy and bevacizumab treatment in IMpower150 (30). The association between EGFR mutation status and atezolizumab therapy response should be explored, and further studies of these mechanisms are needed to effectively predict survival and to provide a better personalized treatment for individuals with EGFR mutations in NSCLC.

Atezolizumab is widely used in clinical practice, and this information is essential to maximize the benefit to individuals with NSCLC. We found that only white patients achieved OS improvement with 1st-line combination therapy based on atezolizumab, while both Asian patients and Black or African Americans did not. In terms of PFS, we found that both Asian and white patients achieved survival improvement, but Black and African Americans did not. Given the analysis of subgroups, the results should be interpreted with caution because of the small number of individuals analyzed. Individuals with advanced NSCLC of different races have different clinical and genetic characteristics and socio-environmental makeup, which may influence their response to atezolizumab (79). It is possible that there is some yet unknown mechanism that could explain these differences, or it is far more likely that this statistical significance is due to chance. Therefore, further research and confirmatory studies with large numbers of patients applying atezolizumab-based therapy in different races, including ≥2nd-line therapy, are needed. Additionally, based on the results of our analysis, race can be used as a suitable predictor of atezolizumab.

The expression of PD-L1 in tumor cells assessed by immunohistochemistry is a crucial means to choose and stratify NSCLC individuals who could show better potency of checkpoint inhibitors (80). PD-L1 expression patterns in TC or IC have been discovered to possess potential clinical and biological relevance in NSCLC, and their expression independently attenuates anti-tumor immune function (80, 81). Our analysis results show that NSCLC patients with PD-L1-negative expression (TC0 and IC0) can have improved OS from atezolizumab, and we recommend that these patients use atezolizumab as a 1st-line monotherapy. Therefore, even for PD-L1-negative individuals, atezolizumab treatment is a good treatment option for such patients. Although individuals with low levels of PD-L1 expression (TC1/2 or IC1/2) could benefit from PFS after atezolizumab-based therapy (HR = 0.66; 95% CI, 0.56–0.77; *p* < 0.00001), there was no significant difference in OS results compared with chemotherapy-based treatment (HR = 0.83; 95% CI, 0.65–1.06; *p* = 0.13), which may be due to the fact that PFS benefits did not translate into OS benefits, which may be an inherent limitation of the experiments involved because of the risk of systematic bias and confounding factors, such as differences in the performance of different PD-L1 assays (82), or may be because PD-L1 expression did not take into account other interferences that inhibit the immune response to tumor cells, such as regulatory lymphocytes and bone marrow-derived suppressor cells (83), all of which resulted in the finding that OS did not benefit in this kind of population. When we performed a sensitivity analysis of patients with TC1/2 or IC1/2, excluding IMpower132, the 1st-line monotherapy based on atezolizumab, we unexpectedly observed a significant OS benefit after applying atezolizumab. Therefore, since there is only one monotherapy study based on atezolizumab and the evidence is insufficient, atezolizumab combination therapy should be prioritized in these patients. Furthermore, according to our results, for NCSLC patients with positive PD-L1 expression (TC1/2/3 or IC1/2/3), although OS was improved regardless of the treatment line and treatment regime, we did not find that atezolizumab, as ≥2nd-line treatment, improved patients’ PFS. Therefore, according to our in-depth analyses of **Tables 2** and **S3**, we strongly recommend 1st-line atezolizumab combination therapy for these patients. Following this, an enhanced OS and PFS benefit was observed in the high-level expression of PD-L1 (TC3 or IC3) regardless of the treatment line or regimen. Studies have shown that atezolizumab has a long-lasting clinical response in individuals with advanced NSCLC with high levels of PD-L1 expression on TC or IC, which supports our findings (80, 81). Our analysis results also showed that TC2/3 or IC2/3 patients with NSCLC could obtain OS improvement from atezolizumab, and we recommended that these patients should use atezolizumab for ≥2nd-line monotherapy. Thus, PD-L1 expression can be considered as a suitable biomarker for atezolizumab-based therapy. A full understanding of the association between PD-L1 expression and the therapeutic effect of atezolizumab is conducive to the better selection of detailed regimens and improvement of the individualization of treatment.

In addition, although our results suggest that the treatment line cannot be used to predict the potency of atezolizumab, both 1st-line and ≥2nd-line treatments achieved OS improvement. However, the ≥2nd-line treatment subgroup seemed to have a better OS benefit (HR, 0.84; 95% CI, 0.77–0.90; for 1st-line; HR, 0.79; 95% CI, 0.70–0.90; for ≥2nd-line therapy). However, previous studies have shown that the treatment line could be deemed an appropriate predictor in PD-1/PD-L1 inhibitor therapy, and the 1st-line treatment subgroup has a better OS benefit. They believe that previous traditional chemotherapy or radiotherapy may produce potential immunosuppression and serious adverse reactions, which may have a negative impact on future immunotherapy (84). Inconsistent with our analysis, these contradictory findings may be the result of a combination of PD-1 and PD-L1 inhibitors, while our results specifically focused on the potency of atezolizumab in individuals receiving different treatment lines. In atezolizumab 1st-line therapy, the coverage of those who benefited from combination therapy was broader, while monotherapy benefits were observed only in patients with PS 0, TC0, and IC0. Some studies have shown that chemotherapy and ICI given at the same time may improve the anti-tumor effect of ICI. The immunogenic effect of chemotherapy is explained by several proposed mechanisms: the cytotoxicity of chemotherapy leads to the shedding of tumor antigen and an increase in dendritic cell antigen cross-presentation, and changes in the immune regulatory system lead to the proliferation of effector T cells, inhibition of regulatory T cells, and enhanced innate immunity (85–88). There are few studies and data related to ≥2nd-line combination therapy and insufficient evidence; therefore, it is worth paying close attention to the efficacy of atezolizumab ≥2nd-line combination therapy in subsequent studies.

It is of great concern to explore the molecular and immune mechanisms of atezolizumab and reveal the reasons why these patients may get or lack benefit from atezolizumab treatment, which is a great challenge for research in the field of cancer treatment in the future and also helpful for personalized treatment in the future. Although our research yielded useful insights, we acknowledge that this study has some limitations. First, these data were extracted from pooled data rather than from individuals in the trials, leading to selection bias. Second, different clinicopathological features, such as smoking status and EGFR mutations, may be associated with each other, causing confounding bias. While we are mainly concerned with a single trait, other confounding variables may affect survival outcomes. Third, since our study was based on correlation rather than causal findings, further research is required to comprehend the mechanisms by which different clinical and molecular features can predict atezolizumab potency, and to determine whether other biomarkers are associated with atezolizumab potency. The open-label design used in the included studies was another limitation, which may have led to potentially biased OS and PFS results.

From our meta-analysis, in NSCLC individuals, age group, smoking status, liver metastasis status, EGFR mutation status, race, as well as expression of PD-L1 can predict the potency of atezolizumab in individuals aged <65 years old and 65–74 years old, active or previous smokers, those without liver metastasis, those with EGFR wild-type, white individuals, and those with TC3 or IC3, TC2/3 or IC2/3, TC1/2/3 or IC1/2/3, as well as TC0 and IC0, all of whom may benefit from atezolizumab treatment. Atezolizumab can improve OS regardless of sex, histological type, ECOG PS performance status, and treatment line. According to subgroup analysis, male individuals, those with non-squamous NSCLC and PS 1, active or previous smokers, and those with wild-type EGFR, TC3 or IC3, and TC1/2/3 or IC1/2/3 could benefit from atezolizumab treatment not related to treatment line and treatment regimen.

In summary, specific clinical characteristics can be used to predict atezolizumab potency. These findings contribute to the practical application of atezolizumab in obtaining more accurate treatments for NSCLC. Subgroup analysis suggests that the appropriate population should be considered when selecting atezolizumab treatment to refine the choice of scheme and improve the individualization of treatment.

## Data Availability Statement

The original contributions presented in the study are included in the article/**Supplementary Material**. Further inquiries can be directed to the corresponding author.

## Author Contributions

Conception and design: PC, WL, and GH. Collection and assembly of data: WL. Data analysis and interpretation: WL. Manuscript writing: WL and GH. Final approval of the manuscript: All authors. All authors contributed to the article and approved the submitted version.

## Funding

This study was supported by funding from the Tianjin Major Disease Prevention and Control Science and Technology Project, Tianjin Municipal Science and Technology Bureau (18ZXDBSY00050).

## Conflict of Interest

The authors declare that the research was conducted in the absence of any commercial or financial relationships that could be construed as a potential conflict of interest.

## Publisher’s Note

All claims expressed in this article are solely those of the authors and do not necessarily represent those of their affiliated organizations, or those of the publisher, the editors and the reviewers. Any product that may be evaluated in this article, or claim that may be made by its manufacturer, is not guaranteed or endorsed by the publisher.
