# Supplementary material for: Efficacy of Atezolizumab for Advanced Non-Small Cell Lung Cancer Based on Clinical and Molecular Features: A Meta-Analysis
Source: Front Immunol. 2022 Jun 21;13:909027. doi: 10.3389/fimmu.2022.909027 (PMC9253603; doi:10.3389/fimmu.2022.909027)
Supplement: Supplementary file 1 [file DataSheet_1.pdf]

Table S1 Analyses of OS in subgroups of patients with different clinical and molecular features

Population	Subgroup	No. of studies	Test of association			Test of heterogeneity	
			HR	95%CI	P value	I^2	P value
Aged <65 years	Total	7	0.82	0.75-0.90	<0.0001	0%	0.98
	1st Line	6	0.82	0.73-0.91	0.0003	0%	0.95
	≥2nd Line	1	0.84	0.70-1.01	0.06		
	monotherapy	3	0.84	0.73-0.97	0.02	0%	0.79
	combination therapy	4	0.81	0.72-0.92	0.0007	0%	0.91
Aged ≥65 years	Total	3	0.78	0.67-0.90	0.0006	0%	0.79
	1st Line	2	0.81	0.66-1.00	0.05	0%	0.71
	≥2nd Line	1	0.75	061-0.91	0.004		
	monotherapy	2	0.78	0.66-0.91	0.002	0%	0.49
	combination therapy	1	0.78	0.58-1.05	0.10		
Aged 65-74 years	Total	4	0.84	0.72-0.98	0.02	0%	0.77
	1st Line	4	0.84	0.72-0.98	0.02	0%	0.77
	≥2nd Line	0					
	monotherapy	1	0.78	0.45-1.36	0.38		
	combination therapy	3	0.84	0.72-0.99	0.04	0%	0.58
Aged ≥75 years	Total	4	0.89	0.66-1.19	0.42	0%	0.72
	1st Line	4	0.89	0.66-1.19	0.42	0%	0.72
	≥2nd Line	0					
	monotherapy	1	1.03	0.31-3.48	0.96		
	combination therapy	3	0.88	0.65-1.19	0.40	0%	0.52
Female	Total	7	0.80	0.71-0.90	0.0002	0%	0.84
	1st Line	6	0.80	0.70-0.91	0.0010	0%	0.74
	≥2nd Line	1	0.81	0.65-1.01	0.06		
	monotherapy	3	0.80	0.67-0.96	0.01	0%	0.95
	combination therapy	4	0.80	0.69-0.93	0.004	0%	0.45
Male	Total	7	0.83	0.76-0.90	<0.0001	0%	0.78
	1st Line	6	0.85	0.77-0.93	0.0007	0%	0.75
	≥2nd Line	1	0.79	0.66-0.93	0.005		
	monotherapy	3	0.82	0.72-0.93	0.003	0%	0.46
	combination therapy	4	0.84	0.75-0.93	0.001	0%	0.66
Non-squamous	Total	7	0.81	0.75-0.88	<0.00001	0%	0.92
	1st Line	5	0.82	0.75-0.90	<0.0001	0%	0.892
	≥2nd Line	2	0.78	0.67-0.90	0.0008	0%	0.51
	monotherapy	4	0.80	0.71-0.90	0.0001	0%	0.70
	combination therapy	3	0.82	0.74-0.91	0.0002	0%	0.79
Squamous	Total	4	0.84	0.74-0.97	0.01	0%	0.92
	1st Line	2	0.88	0.74-1.05	0.15	0%	0.92
	≥2nd Line	2	0.79	0.64-0.99	0.04	0%	0.98
	monotherapy	3	0.80	0.65-0.99	0.04	0%	0.94
	combination therapy	1	0.88	0.73-1.05	0.15		
PS 0	Total	7	0.82	0.73-0.92	0.0008	0%	0.93
	1st Line	6	0.83	0.72-0.94	0.004	0%	0.86
	≥2nd Line	1	0.80	0.63-1.02	0.07		
	monotherapy	3	0.77	0.64-0.93	0.006	0%	0.78
	combination therapy	4	0.85	0.74-0.99	0.04	0%	0.87
PS 1	Total	7	0.81	0.74-0.88	<0.00001	0%	0.66
	1st Line	6	0.83	0.75-0.91	0.0002	0%	0.62

	≥2nd Line	1	0.77	0.65-0.90	0.001		
	monotherapy	3	0.82	0.72-0.93	0.003	25%	0.26
	combination therapy	4	0.80	0.72-0.90	<0.0001	0%	0.71
Active or previous smoker	Total	7	0.82	0.76-0.88	<0.00001	0%	0.94
	1st Line	5	0.84	0.77-0.92	<0.0001	0%	0.97
	≥2nd Line	2	0.77	0.67-0.88	0.0002	0%	0.85
	monotherapy	3	0.80	0.72-0.90	0.0002	0%	0.54
	combination therapy	4	0.83	0.76-0.92	0.0002	0%	0.97
Never smoker	Total	8	0.84	0.70-1.00	0.05	0%	0.51
	1st Line	6	0.83	0.68-1.03	0.09	1%	0.41
	≥2nd Line	2	0.85	0.62-1.16	0.31	20%	0.26
	monotherapy	4	0.89	0.68-1.17	0.41	30%	0.23
	combination therapy	4	0.80	0.64-1.01	0.06	0%	0.65
With liver metastases	Total	5	0.94	0.78-1.14	0.55	0%	0.49
	1st Line	5	0.94	0.78-1.14	0.55	0%	0.49
	≥2nd Line	0					
	monotherapy	1	0.96	0.58-1.58	0.86		
	combination therapy	4	0.94	0.77-1.16	0.57	12%	0.33
Without liver metastases	Total	3	0.80	0.70-0.90	0.0005	0%	0.60
	1st Line	3	0.80	0.70-0.90	0.0005	0%	0.60
	≥2nd Line	0					
	monotherapy	1	0.86	0.69-1.06	0.15		
	combination therapy	2	0.77	0.66-0.90	0.0010	0%	0.54
EGFR mutant	Total	3	1.09	0.81-1.47	0.56	0%	0.75
	1st Line	2	1.04	0.72-1.50	0.82	0%	0.53
	≥2nd Line	1	1.19	0.72-1.97	0.50		
	monotherapy	1	1.19	0.72-1.97	0.50		
	combination therapy	2	1.04	0.72-1.50	0.82	0%	0.53
EGFR wildtype	Total	3	0.80	0.72-0.88	<0.00001	0%	0.69
	1st Line	2	0.82	0.73-0.93	0.002	0%	0.66
	≥2nd Line	1	0.76	0.65-0.89	0.0006		
	monotherapy	1	0.76	0.65-0.89	0.0006		
	combination therapy	2	0.82	0.73-0.93	0.002	0%	0.66
Asian	Total	5	0.89	0.70-1.15	0.38	17%	0.30
	1st Line	5	0.89	0.70-1.15	0.38	17%	0.30
	≥2nd Line	0					
	monotherapy	2	0.66	0.43-1.01	0.05	5%	0.30
	combination therapy	3	1.05	0.77-1.44	0.74	0%	0.70
Black or African American	Total	3	0.84	0.36-1.95	0.68	0%	0.83
	1st Line	3	0.84	0.36-1.95	0.68	0%	0.83
	≥2nd Line	0					
	monotherapy	0					
	combination therapy	3	0.84	0.36-1.95	0.68	0%	0.83
White	Total	5	0.84	0.76-0.92	0.0002	0%	0.92
	1st Line	5	0.84	0.76-0.92	0.0002	0%	0.92
	≥2nd Line	0					
	monotherapy	2	0.89	0.73-1.09	0.25	0%	0.82
	combination therapy	3	0.82	0.74-0.91	0.0003	0%	0.83

TC0 and IC0	Total	7	0.87	0.78-0.96	0.005	0%	0.73
	1st Line	5	0.86	0.77-0.97	0.02	0%	0.55
	≥2nd Line	2	0.87	0.72-1.04	0.13	0%	0.46
	monotherapy	3	0.82	0.69-0.97	0.02	6%	0.35
	combination therapy	4	0.89	0.79-1.01	0.08	0%	0.83
TC1/2/3 or IC1/2/3	Total	6	0.77	0.70-0.85	<0.00001	0%	0.52
	1st Line	4	0.79	0.70-0.89	0.0001	0%	0.57
	≥2nd Line	2	0.73	0.62-0.86	0.0002	37%	0.21
	monotherapy	3	0.77	0.68-0.88	<0.0001	30%	0.24
	combination therapy	3	0.76	0.66-0.88	0.0003	0%	0.51
TC1/2 or IC1/2	Total	5	0.83	0.65-1.06	0.13	64%	0.02
	1st Line	5	0.83	0.65-1.06	0.13	64%	0.02
	≥2nd Line	0					
	monotherapy	1	1.18	0.80-1.76	0.40		
	combination therapy	4	0.77	0.60-0.98	0.04	59%	0.06
TC2/3 or IC2/3	Total	4	0.72	0.61-0.84	<0.0001	20%	0.29
	1st Line	2	0.80	0.65-0.99	0.04	0%	0.40
	≥2nd Line	2	0.62	0.49-0.78	<0.0001	0%	0.56
	monotherapy	3	0.71	0.60-0.85	0.0002	47%	0.15
	combination therapy	1	0.72	0.52-1.00	0.05		
TC3 or IC3	Total	8	0.65	0.55-0.76	<0.00001	4%	0.40
	1st Line	6	0.71	0.59-0.86	0.0005	0%	0.73
	≥2nd Line	2	0.49	0.35-0.67	<0.00001	0%	0.58
	monotherapy	4	0.60	0.48-0.76	<0.0001	25%	0.26
	combination therapy	4	0.69	0.55-0.87	0.002	0%	0.46

Table S2 Analyses of PFS in subgroups of patients with different clinical and molecular features

Population	Subgroup	No. of studies	Test of association			Test of heterogeneity	
			HR	95%CI	P value	I^2	P value
Aged <65 years	Total	4	0.67	0.59-0.76	<0.00001	0%	0.60
	1st Line	4	0.67	0.59-0.76	<0.00001	0%	0.60
	≥2nd Line	0					
	monotherapy	1	0.63	0.49-0.80	0.0002		
	combination therapy	3	0.68	0.59-0.79	<0.00001	0%	0.48
Aged ≥65 years	Total	2	0.60	0.50-0.72	<0.00001	0%	0.44
	1st Line	2	0.60	0.50-0.72	<0.00001	0%	0.44
	≥2nd Line	0					
	monotherapy	1	0.55	0.42-0.73	<0.0001		
	combination therapy	1	0.64	0.50-0.82	0.0004		
Aged 65-74 years	Total	2	0.60	0.49-0.73	<0.00001	26%	0.24
	1st Line	2	0.60	0.49-0.73	<0.00001	26%	0.24
	≥2nd Line	0					
	monotherapy	0					
	combination therapy	2	0.60	0.49-0.73	<0.00001	26%	0.24
Aged ≥75 years	Total	2	0.62	0.43-0.90	0.01	22%	0.26
	1st Line	2	0.62	0.43-0.90	0.01	22%	0.26
	≥2nd Line	0					
	monotherapy	0					
	combination therapy	2	0.62	0.43-0.90	0.01	22%	0.26
Female	Total	4	0.62	0.53-0.73	<0.00001	0%	0.42
	1st Line	4	0.62	0.53-0.73	<0.00001	0%	0.42
	≥2nd Line	0					
	monotherapy	1	0.51	0.36-0.71	<0.0001		
	combination therapy	3	0.66	0.55-0.78	<0.00001	0%	0.59
Male	Total	4	0.64	0.58-0.72	<0.00001	11%	0.34
	1st Line	4	0.64	0.58-0.72	<0.00001	11%	0.34
	≥2nd Line	0					
	monotherapy	1	0.64	0.51-0.79	<0.0001		
	combination therapy	3	0.65	0.57-0.73	<0.00001	40%	0.19
Non-squamous	Total	3	0.61	0.55-0.67	<0.00001	0%	0.73
	1st Line	3	0.61	0.55-0.67	<0.00001	0%	0.73
	≥2nd Line	0					
	monotherapy	1	0.60	0.49-0.72	<0.00001		
	combination therapy	2	0.61	0.54-0.69	<0.00001	0%	0.45
Squamous	Total	2	0.80	0.62-1.05	0.11	65%	0.99
	1st Line	1	0.71	0.60-0.85	0.0002		
	≥2nd Line	1	0.94	0.72-1.23	0.66		
	monotherapy	1	0.94	0.72-1.23	0.66		
	combination therapy	1	0.71	0.60-0.85	0.0002		
PS 0	Total	4	0.59	0.51-0.69	<0.00001	0%	0.74
	1st Line	4	0.59	0.51-0.69	<0.00001	0%	0.74
	≥2nd Line	0					
	monotherapy	1	0.56	0.42-0.76	0.0002		
	combination therapy	3	0.60	0.51-0.71	<0.00001	0%	0.58
PS 1	Total	4	0.66	0.59-0.74	<0.00001	0%	0.88
	1st Line	4	0.66	0.59-0.74	<0.00001	0%	0.88

	≥2nd Line	0					
	monotherapy	1	0.63	0.49-0.79	0.0001		
	combination therapy	3	0.67	0.59-0.76	<0.00001	0%	0.82
Active or previous smoker	Total	4	0.63	0.58-0.70	<0.00001	0%	0.51
	1st Line	4	0.63	0.58-0.70	<0.00001	0%	0.51
	≥2nd Line	0					
	monotherapy	1	0.61	0.50-0.74	<0.00001		
	combination therapy	3	0.64	0.58-0.71	<0.00001	5%	0.35
Never smoker	Total	4	0.68	0.52-0.89	0.004	0%	0.57
	1st Line	4	0.68	0.52-0.89	0.004	0%	0.57
	≥2nd Line	0					
	monotherapy	1	0.49	0.28-0.87	0.01		
	combination therapy	3	0.74	0.55-1.00	0.05	0%	0.79
With liver metastases	Total	5	0.71	0.54-0.94	0.02	54%	0.07
	1st Line	5	0.71	0.54-0.94	0.02	54%	0.07
	≥2nd Line	0					
	monotherapy	1	0.77	0.47-1.25	0.29		
	combination therapy	4	0.70	0.50-0.99	0.04	65%	0.04
Without liver metastases	Total	4	0.62	0.56-0.68	<0.00001	0%	0.57
	1st Line	4	0.62	0.56-0.68	<0.00001	0%	0.57
	≥2nd Line	0					
	monotherapy	1	0.56	0.46-0.69	<0.00001		
	combination therapy	3	0.63	0.57-0.71	<0.00001	0%	0.59
EGFR mutant	Total	2	0.85	0.46-1.56	0.60	69%	0.07
	1st Line	2	0.85	0.46-1.56	0.60	69%	0.07
	≥2nd Line	0					
	monotherapy	0					
	combination therapy	2	0.85	0.46-1.56	0.60	69%	0.07
EGFR wildtype	Total	1	0.62	0.52-0.74	<0.00001		
	1st Line	1	0.62	0.52-0.74	<0.00001		
	≥2nd Line	0					
	monotherapy	0					
	combination therapy	1	0.62	0.52-0.74	<0.00001		
Asian	Total	2	0.53	0.32-0.86	0.010	56%	0.13
	1st Line	2	0.53	0.32-0.86	0.010	56%	0.13
	≥2nd Line	0					
	monotherapy	1	0.42	0.28-0.63	<0.0001		
	combination therapy	1	0.69	0.42-1.15	0.16		
Black or African American	Total	1	0.39	0.07-2.08	0.27		
	1st Line	1	0.39	0.07-2.08	0.27		
	≥2nd Line	0					
	monotherapy	0					
	combination therapy	1	0.39	0.07-2.08	0.27		
White	Total	2	0.69	0.60-0.79	<0.00001	0%	0.76
	1st Line	2	0.69	0.60-0.79	<0.00001	0%	0.76
	≥2nd Line	0					
	monotherapy	1	0.67	0.54-0.84	0.0005		
	combination therapy	1	0.70	0.59-0.84	<0.0001		
TC0 and IC0	Total	6	0.80	0.63-1.01	0.07	80%	0.0002

	1st Line	4	0.69	0.56-0.86	0.0009	63%	0.04
	≥2nd Line	2	1.12	0.94-1.32	0.21	0%	0.97
	monotherapy	3	0.83	0.46-1.50	0.53	90%	<0.0001
	combination therapy	3	0.77	0.67-0.88	0.0002	0%	0.77
TC1/2/3 or IC1/2/3	Total	5	0.70	0.57-0.85	0.0003	75%	0.003
	1st Line	3	0.61	0.49-0.75	<0.00001	64%	0.06
	≥2nd Line	2	0.86	0.75-1.00	0.05	0%	0.94
	monotherapy	3	0.80	0.72-0.90	0.0001	18%	0.29
	combination therapy	2	0.55	0.47-0.66	<0.00001	21%	0.26
TC1/2 or IC1/2	Total	4	0.66	0.56-0.77	<0.00001	0%	0.47
	1st Line	4	0.66	0.56-0.77	<0.00001	0%	0.47
	≥2nd Line	0					
	monotherapy	1	0.80	0.56-1.16	0.24		
	combination therapy	3	0.63	0.53-0.75	<0.00001	0%	0.58
TC2/3 or IC2/3	Total	4	0.65	0.57-0.75	<0.00001	0%	0.47
	1st Line	2	0.60	0.49-0.72	<0.00001	0%	0.39
	≥2nd Line	2	0.72	0.59-0.89	0.002	0%	0.97
	monotherapy	3	0.69	0.59-0.81	<0.00001	0%	0.75
	combination therapy	1	0.54	0.40-0.73	<0.0001		
TC3 or IC3	Total	7	0.52	0.44-0.61	<0.00001	0%	0.73
	1st Line	5	0.50	0.41-0.60	<0.00001	0%	0.61
	≥2nd Line	2	0.59	0.43-0.81	0.001	0%	0.96
	monotherapy	4	0.58	0.47-0.72	<0.00001	0%	0.94
	combination therapy	3	0.45	0.35-0.58	<0.00001	0%	0.66

Table S3 Analyses of OS in subgroups of patients with different clinical features and treatment regimens

Population	Subgroup	Regimen	No. of studies	Test of association			Test of heterogeneity	
				HR	95%CI	P value	I^2	P value
Aged <65 years	1st Line	combined	4	0.81	0.72-0.92	0.0007	0%	0.91
		mono	2	0.84	0.66-1.07	0.16	0%	0.50
	≥2nd Line	mono	1	0.84	0.70-1.01	0.06		
Aged ≥65 years	1st Line	combined	1	0.78	0.58-1.05	0.10		
		mono	1	0.84	0.63-1.13	0.25		
	≥2nd Line	mono	1	0.75	0.61-0.91	0.004		
Aged 65-74 years	1st Line	combined	3	0.84	0.72-0.99	0.04	0%	0.58
		mono	1	0.78	0.45-1.36	0.38		
	≥2nd Line	mono	0					
Aged ≥75 years	1st Line	combined	3	0.88	0.65-1.19	0.40	0%	0.52
		mono	1	1.03	0.31-3.48	0.96		
	≥2nd Line	mono	0					
Female	1st Line	combined	4	0.80	0.69-0.93	0.004	0%	0.45
		mono	2	0.78	0.58-1.06	0.12	0%	0.79
	≥2nd Line	mono	1	0.81	0.65-1.01	0.06		
Male	1st Line	combined	4	0.84	0.75-0.93	0.001	0%	0.66
		mono	2	0.87	0.71-1.08	0.21	0%	0.33
	≥2nd Line	mono	1	0.79	0.66-0.93	0.005		
Non-squamous	1st Line	combined	3	0.82	0.74-0.91	0.0002	0%	0.79
		mono	2	0.83	0.70-1.00	0.05	0%	0.43
	≥2nd Line	mono	2	0.78	0.67-0.90	0.0008	0%	0.51
Squamous	1st Line	combined	1	0.88	0.73-1.05	0.15		
		mono	1	0.91	0.45-1.83	0.79		
	≥2nd Line	mono	2	0.79	0.64-0.99	0.04	0%	0.98
PS 0	1st Line	combined	4	0.85	0.74-0.99	0.04	0%	0.87
		mono	2	0.73	0.55-0.97	0.03	0%	0.62
	≥2nd Line	mono	1	0.80	0.63-1.02	0.07		
PS 1	1st Line	combined	4	0.80	0.72-0.90	<0.0001	0%	0.71
		mono	2	0.93	0.75-1.16	0.53	0%	0.41
	≥2nd Line	mono	1	0.77	0.65-0.90	0.001		
Active or previous smoker	1st Line	combined	4	0.83	0.76-0.92	0.0002	0%	0.97
		mono	1	0.89	0.72-1.09	0.25		
	≥2nd Line	mono	2	0.77	0.67-0.88	0.0002	0%	0.85
Never smoker	1st Line	combined	4	0.80	0.64-1.01	0.06	0%	0.65
		mono	2	1.15	0.47-2.85	0.76	62%	0.10
	≥2nd Line	mono	2	0.85	0.62-1.16	0.31	20%	0.26
With liver metastases	1st Line	combined	4	0.94	0.77-1.16	0.57	12%	0.33
		mono	1	0.96	0.58-1.58	0.86		
	≥2nd Line	mono	0					
Without liver metastases	1st Line	combined	2	0.77	0.66-0.90	0.0010	0%	0.54
		mono	1	0.86	0.69-1.06	0.15		
	≥2nd Line	mono	0					
EGFR mutant	1st Line	combined	2	1.04	0.72-1.50	0.82	0%	0.53
		mono	0					
	≥2nd Line	mono	1	1.19	0.72-1.97	0.50		
EGFR wildtype	1st Line	combined	2	0.82	0.73-0.93	0.002	0%	0.66
		mono	0					

	≥2nd Line	mono	1	0.76	0.65-0.89	0.0006		
Asian	1st Line	combined	3	1.05	0.77-1.44	0.74	0%	0.70
		mono	2	0.66	0.43-1.01	0.05	5%	0.30
	≥2nd Line	mono	0					
Black or African American	1st Line	combined	3	0.84	0.36-1.95	0.68	0%	0.83
		mono	0					
	≥2nd Line	mono	0					
White	1st Line	combined	3	0.82	0.74-0.91	0.0003	0%	0.83
		mono	2	0.89	0.73-1.09	0.25	0%	0.82
	≥2nd Line	mono	0					
TC0 and IC0	1st Line	combined	4	0.89	0.79-1.01	0.08	0%	0.83
		mono	1	0.67	0.46-0.96	0.03		
	≥2nd Line	mono	2	0.87	0.72-1.04	0.13	0%	0.46
TC1/2/3 or IC1/2/3	1st Line	combined	3	0.76	0.66-0.88	0.0003	0%	0.51
		mono	1	0.85	0.69-1.04	0.11		
	≥2nd Line	mono	2	0.73	0.62-0.86	0.0002	37%	0.21
TC1/2 or IC1/2	1st Line	combined	4	0.77	0.60-0.98	0.04	59%	0.06
		mono	1	1.18	0.80-1.76	0.40		
	≥2nd Line	mono	0					
TC2/3 or IC2/3	1st Line	combined	1	0.72	0.52-1.00	0.05		
		mono	1	0.87	0.66-1.14	0.31		
	≥2nd Line	mono	2	0.62	0.49-0.78	<0.0001	0%	0.56
TC3 or IC3	1st Line	combined	4	0.69	0.55-0.87	0.002	0%	0.46
		mono	2	0.76	0.55-1.05	0.10	0%	0.93
	≥2nd Line	mono	2	0.49	0.35-0.67	<0.00001	0%	0.58

Table S4 Analyses of PFS in subgroups of patients with different clinical features and treatment regimens

Population	Subgroup	Regimen	No. of studies	Test of association			Test of heterogeneity	
				HR	95%CI	P value	I^2	P value
Aged <65 years	1st Line	combined	3	0.68	0.59-0.79	<0.00001	0%	0.48
		mono	1	0.63	0.49-0.80	0.0002		
	≥2nd Line	mono	0					
Aged ≥65 years	1st Line	combined	1	0.64	0.50-0.82	0.0004		
		mono	1	0.55	0.42-0.73	<0.0001		
	≥2nd Line	mono	0					
Aged 65-74 years	1st Line	combined	2	0.60	0.49-0.73	<0.00001	26%	0.24
		mono	0					
	≥2nd Line	mono	0					
Aged ≥75 years	1st Line	combined	2	0.62	0.43-0.90	0.01	22%	0.26
		mono	0					
	≥2nd Line	mono	0					
Female	1st Line	combined	3	0.66	0.55-0.78	<0.00001	0%	0.59
		mono	1	0.51	0.36-0.71	<0.0001		
	≥2nd Line	mono	0					
Male	1st Line	combined	3	0.65	0.57-0.73	<0.00001	40%	0.19
		mono	1	0.64	0.51-0.79	<0.0001		
	≥2nd Line	mono	0					
Non-squamous	1st Line	combined	2	0.61	0.54-0.69	<0.00001	0%	0.45
		mono	1	0.60	0.49-0.72	<0.00001		
	≥2nd Line	mono	0					
Squamous	1st Line	combined	1	0.71	0.60-0.85	0.0002		
		mono	0					
	≥2nd Line	mono	1	0.94	0.72-1.23	0.66		
PS 0	1st Line	combined	3	0.60	0.51-0.71	<0.00001	0%	0.58
		mono	1	0.56	0.42-0.76	0.0002		
	≥2nd Line	mono	0					
PS 1	1st Line	combined	3	0.67	0.59-0.76	<0.00001	0%	0.82
		mono	1	0.63	0.49-0.79	0.0001		
	≥2nd Line	mono	0					
Active or previous smoker	1st Line	combined	3	0.64	0.58-0.71	<0.00001	5%	0.35
		mono	1	0.61	0.50-0.74	<0.00001		
	≥2nd Line	mono	0					
Never smoker	1st Line	combined	3	0.74	0.55-1.00	0.05	0%	0.79
		mono	1	0.49	0.28-0.87	0.01		
	≥2nd Line	mono	0					
With liver metastases	1st Line	combined	4	0.70	0.50-0.99	0.04	65%	0.04
		mono	1	0.77	0.47-1.25	0.29		
	≥2nd Line	mono	0					
Without liver metastases	1st Line	combined	3	0.63	0.57-0.71	<0.00001	0%	0.59
		mono	1	0.56	0.46-0.69	<0.00001		
	≥2nd Line	mono	0					
EGFR mutant	1st Line	combined	2	0.85	0.46-1.56	0.60	69%	0.07
		mono	0					
	≥2nd Line	mono	0					
EGFR wildtype	1st Line	combined	1	0.62	0.52-0.74	<0.00001		

		mono	0					
	≥2nd Line	mono	0					
Asian	1st Line	combined	1	0.69	0.42-1.15	0.16		
		mono	1	0.42	0.28-0.63	<0.0001		
	≥2nd Line	mono	0					
Black or African American	1st Line	combined	1	0.39	0.07-2.08	0.27		
		mono	0					
	≥2nd Line	mono	0					
White	1st Line	combined	1	0.70	0.59-0.84	<0.0001		
		mono	1	0.67	0.54-0.84	0.0005		
	≥2nd Line	mono	0					
TC0 and IC0	1st Line	combined	3	0.77	0.67-0.88	0.0002	0%	0.77
		mono	1	0.45	0.31-0.64	<0.0001		
	≥2nd Line	mono	2	1.12	0.94-1.32	0.21	0%	0.97
TC1/2/3 or IC1/2/3	1st Line	combined	2	0.55	0.47-0.66	<0.00001	21%	0.26
		mono	1	0.72	0.60-0.86	0.0003		
	≥2nd Line	mono	2	0.86	0.75-1.00	0.05	0%	0.94
TC1/2 or IC1/2	1st Line	combined	3	0.63	0.53-0.75	<0.00001	0%	0.58
		mono	1	0.80	0.56-1.16	0.24		
	≥2nd Line	mono	0					
TC2/3 or IC2/3	1st Line	combined	1	0.54	0.40-0.73	<0.0001		
		mono	1	0.64	0.50-0.82	0.0004		
	≥2nd Line	mono	2	0.69	0.59-0.81	<0.00001	0%	0.75
TC3 or IC3	1st Line	combined	3	0.45	0.35-0.58	<0.00001	0%	0.66
		mono	2	0.57	0.42-0.76	0.0001	0%	0.54
	≥2nd Line	mono	2	0.59	0.43-0.81	0.001	0%	0.96

Table S5 Different treatment lines and regimens with PFS benefited from atezolizumab-based therapy over chemotherapy-basedtherapy in targeted patients

Line	Regimen	Population	No.of studies	HR	CI95%	P value
1st Line	combined therapy	Aged <65 years	3	0.68	0.59-0.79	<0.00001
		Aged ≥ 65 years	1	0.64	0.50-0.82	0.0004
		Aged 65-74 years	2	0.60	0.49-0.73	<0.00001
		Aged ≥75 years	2	0.62	0.43-0.90	0.01
		Female	3	0.66	0.55-0.78	<0.00001
		Male	3	0.65	0.57-0.73	<0.00001
		Non-squamous	2	0.61	0.54-0.69	<0.00001
		Squamous	1	0.71	0.60-0.85	0.0002
		PS 0	3	0.60	0.51-0.71	<0.00001
		PS 1	3	0.67	0.59-0.76	<0.00001
		Active or previous smoker	3	0.64	0.58-0.71	<0.00001
		With liver metastases	4	0.70	0.50-0.99	0.04
		Without liver metastases	3	0.63	0.57-0.71	<0.00001
		EGFR wild-type	1	0.62	0.52-0.74	<0.00001
		White	1	0.70	0.59-0.84	<0.0001
		TC0 and IC0	3	0.77	0.67-0.88	0.0002
		TC1/2/3 or IC1/2/3	2	0.55	0.47-0.66	<0.00001
		TC1/2 or IC1/2	3	0.63	0.53-0.75	<0.00001
		TC2/3 or IC2/3	1	0.54	0.40-0.73	<0.0001
		TC3 or IC3	3	0.45	0.35-0.58	<0.00001
	monotherapy	Aged <65 years	1	0.63	0.49-0.80	0.0002
		Aged ≥65 years	1	0.55	0.42-0.73	<0.0001
		Female	1	0.51	0.36-0.71	<0.0001
		Male	1	0.64	0.51-0.79	<0.0001
		Non-squamous	1	0.60	0.49-0.72	<0.00001
		PS 0	1	0.56	0.42-0.76	0.0002
		PS 1	1	0.63	0.49-0.79	0.0001
		Active or previous smoker	1	0.61	0.50-0.74	<0.00001
		Never smoker	1	0.49	0.28-0.87	0.01
		Without liver metastases	1	0.56	0.46-0.69	<0.00001
		Asian	1	0.42	0.28-0.63	<0.0001
		White	1	0.67	0.54-0.84	0.0005
		TC0 and IC0	1	0.45	0.31-0.64	<0.0001
		TC1/2/3 or IC1/2/3	1	0.72	0.60-0.86	0.0003
		TC2/3 or IC2/3	1	0.64	0.50-0.82	0.0004
		TC3 or IC3	2	0.57	0.42-0.76	0.0001
≥2nd Line	monotherapy	TC2/3 or IC2/3	2	0.69	0.59-0.81	<0.00001
		TC3 or IC3	2	0.59	0.43-0.81	0.001

A

	Random sequence generation (selection bias)	Allocation concealment (selection bias)	Blinding of participants and personnel (performance bias)	Blinding of outcome assessment (detection bias)	Incomplete outcome data (attrition bias)	Selective reporting (reporting bias)	Other bias
Fehrenbacher 2016 POPLAR	+	+	−	?	+	+	+
Fehrenbacher 2018 OAK	+	+	−	?	+	+	+
Herbst 2020 IMpower110	+	+	−	?	+	+	+
Jassem 2021 IMpower110	+	+	−	?	+	+	+
Jotte 2020 IMpower131	+	+	−	?	+	+	+
Nishio 2020 IMpower132	+	+	−	?	+	+	+
Nogami 2021 IMpower150 (ABCP vs BCP)	+	+	−	?	+	+	+
Nogami 2021 IMpower150 (ACP vs BCP)	+	+	−	?	+	+	+
Reck 2019 IMpower150 (ABCP vs BCP)	+	+	−	?	+	+	+
Reck 2019 IMpower150 (ACP vs BCP)	+	+	−	?	+	+	+
Rittmeyer 2016 OAK	+	+	−	?	+	+	+
Socinski 2018 IMpower150 (ABCP vs BCP)	+	+	−	?	+	+	+
Socinski 2021 IMpower150 (ABCP vs BCP)	+	+	−	?	+	+	+
Socinski 2021 IMpower150 (ACP vs BCP)	+	+	−	?	+	+	+
West 2019 IMpower130	+	+	−	?	+	+	?

Figure S1: Risk of bias summary.

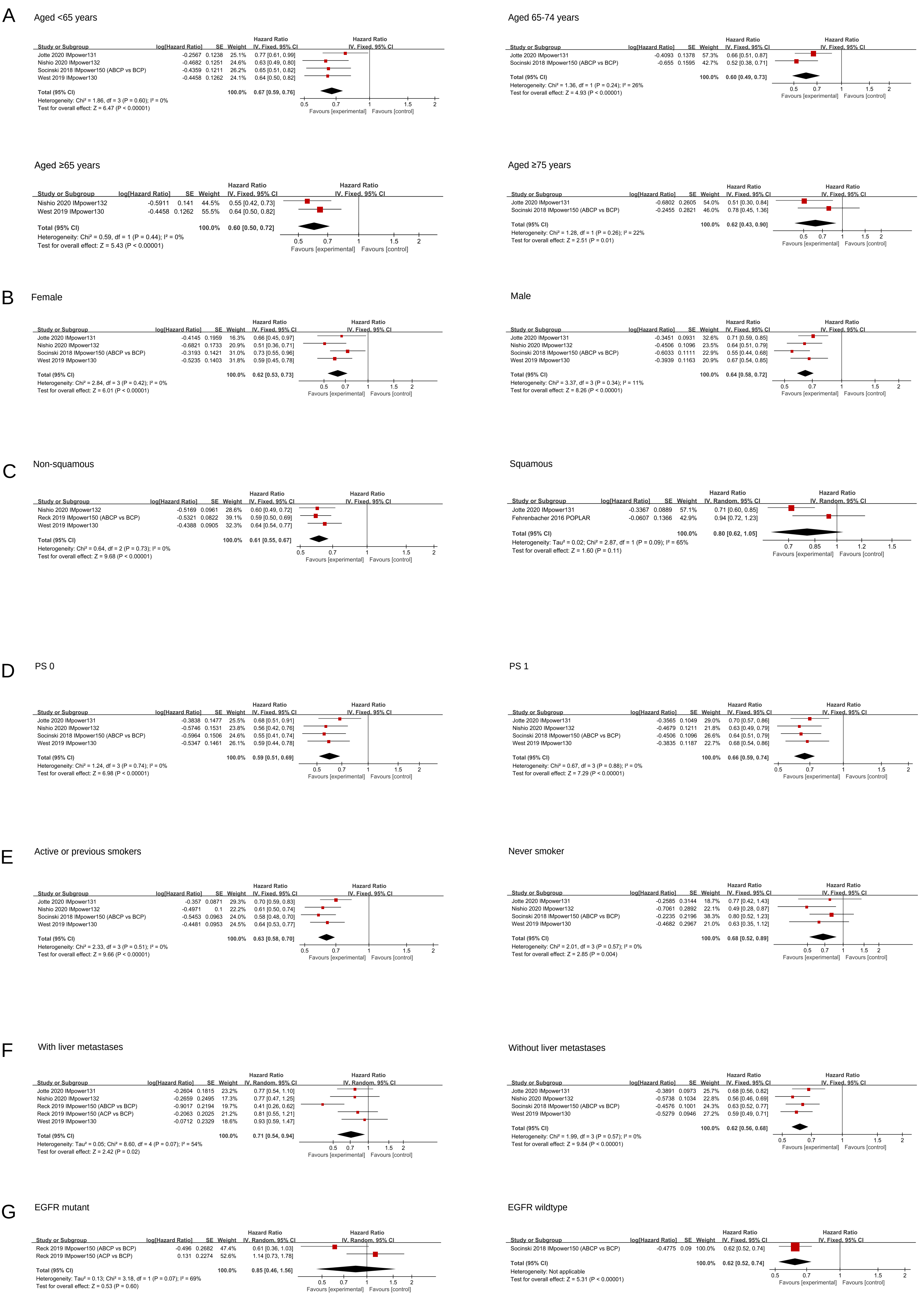
A
Aged <65 years
Study or Subgroup log[Hazard Ratio] SE Weight Hazard Ratio IV, Fixed, 95% CI Hazard Ratio IV, Fixed, 95% CI
Jotte 2020 IMpower131 -0.2567 0.1238 25.1% 0.77 [0.61, 0.99]
Nishio 2020 IMpower132 -0.4682 0.1251 24.6% 0.63 [0.49, 0.80]
Socinski 2018 IMpower150 (ABCP vs BCP) -0.4359 0.1211 26.2% 0.65 [0.51, 0.82]
West 2019 IMpower130 -0.4458 0.1262 24.1% 0.64 [0.50, 0.82]
Total (95% CI) 100.0% 0.67 [0.59, 0.76]
Heterogeneity: Chi² = 1.86, df = 3 (P = 0.60); I² = 0%
Test for overall effect: Z = 6.47 (P < 0.00001)
0.5 0.7 1 1.5 2
Favours [experimental] Favours [control]
Aged 65-74 years
Study or Subgroup log[Hazard Ratio] SE Weight Hazard Ratio IV, Fixed, 95% CI Hazard Ratio IV, Fixed, 95% CI
Jotte 2020 IMpower131 -0.4093 0.1378 57.3% 0.66 [0.51, 0.87]
Socinski 2018 IMpower150 (ABCP vs BCP) -0.655 0.1595 42.7% 0.52 [0.38, 0.71]
Total (95% CI) 100.0% 0.60 [0.49, 0.73]
Heterogeneity: Chi² = 1.36, df = 1 (P = 0.24); I² = 26%
Test for overall effect: Z = 4.93 (P < 0.00001)
0.5 0.7 1 1.5 2
Favours [experimental] Favours [control]
Aged ≥65 years
Study or Subgroup log[Hazard Ratio] SE Weight Hazard Ratio IV, Fixed, 95% CI Hazard Ratio IV, Fixed, 95% CI
Nishio 2020 IMpower132 -0.5911 0.141 44.5% 0.55 [0.42, 0.73]
West 2019 IMpower130 -0.4458 0.1262 55.5% 0.64 [0.50, 0.82]
Total (95% CI) 100.0% 0.60 [0.50, 0.72]
Heterogeneity: Chi² = 0.59, df = 1 (P = 0.44); I² = 0%
Test for overall effect: Z = 5.43 (P < 0.00001)
0.5 0.7 1 1.5 2
Favours [experimental] Favours [control]
Aged ≥75 years
Study or Subgroup log[Hazard Ratio] SE Weight Hazard Ratio IV, Fixed, 95% CI Hazard Ratio IV, Fixed, 95% CI
Jotte 2020 IMpower131 -0.6802 0.2605 54.0% 0.51 [0.30, 0.84]
Socinski 2018 IMpower150 (ABCP vs BCP) -0.2455 0.2821 46.0% 0.78 [0.45, 1.36]
Total (95% CI) 100.0% 0.62 [0.43, 0.90]
Heterogeneity: Chi² = 1.28, df = 1 (P = 0.26); I² = 22%
Test for overall effect: Z = 2.51 (P = 0.01)
0.5 0.7 1 1.5 2
Favours [experimental] Favours [control]
B
Female
Study or Subgroup log[Hazard Ratio] SE Weight Hazard Ratio IV, Fixed, 95% CI Hazard Ratio IV, Fixed, 95% CI
Jotte 2020 IMpower131 -0.4145 0.1959 16.3% 0.66 [0.45, 0.97]
Nishio 2020 IMpower132 -0.6821 0.1733 20.9% 0.51 [0.36, 0.71]
Socinski 2018 IMpower150 (ABCP vs BCP) -0.3193 0.1421 31.0% 0.73 [0.55, 0.96]
West 2019 IMpower130 -0.5235 0.1403 31.8% 0.59 [0.45, 0.78]
Total (95% CI) 100.0% 0.62 [0.53, 0.73]
Heterogeneity: Chi² = 2.84, df = 3 (P = 0.42); I² = 0%
Test for overall effect: Z = 6.01 (P < 0.00001)
0.5 0.7 1 1.5 2
Favours [experimental] Favours [control]
Male
Study or Subgroup log[Hazard Ratio] SE Weight Hazard Ratio IV, Fixed, 95% CI Hazard Ratio IV, Fixed, 95% CI
Jotte 2020 IMpower131 -0.3451 0.0931 32.6% 0.71 [0.59, 0.85]
Nishio 2020 IMpower132 -0.4506 0.1096 23.5% 0.64 [0.51, 0.79]
Socinski 2018 IMpower150 (ABCP vs BCP) -0.6033 0.1111 22.9% 0.55 [0.44, 0.68]
West 2019 IMpower130 -0.3939 0.1163 20.9% 0.67 [0.54, 0.85]
Total (95% CI) 100.0% 0.64 [0.58, 0.72]
Heterogeneity: Chi² = 3.37, df = 3 (P = 0.34); I² = 11%
Test for overall effect: Z = 8.26 (P < 0.00001)
0.5 0.7 1 1.5 2
Favours [experimental] Favours [control]
C
Non-squamous
Study or Subgroup log[Hazard Ratio] SE Weight Hazard Ratio IV, Fixed, 95% CI Hazard Ratio IV, Fixed, 95% CI
Nishio 2020 IMpower132 -0.5169 0.0961 28.6% 0.60 [0.49, 0.72]
Reck 2019 IMpower150 (ABCP vs BCP) -0.5321 0.0822 39.1% 0.59 [0.50, 0.69]
West 2019 IMpower130 -0.4388 0.0905 32.3% 0.64 [0.54, 0.77]
Total (95% CI) 100.0% 0.61 [0.55, 0.67]
Heterogeneity: Chi² = 0.64, df = 2 (P = 0.73); I² = 0%
Test for overall effect: Z = 9.68 (P < 0.00001)
0.5 0.7 1 1.5 2
Favours [experimental] Favours [control]
Squamous
Study or Subgroup log[Hazard Ratio] SE Weight Hazard Ratio IV, Random, 95% CI Hazard Ratio IV, Random, 95% CI
Jotte 2020 IMpower131 -0.3367 0.0889 57.1% 0.71 [0.60, 0.85]
Fehrenbacher 2016 POPLAR -0.0607 0.1366 42.9% 0.94 [0.72, 1.23]
Total (95% CI) 100.0% 0.80 [0.62, 1.05]
Heterogeneity: Tau² = 0.02; Chi² = 2.87, df = 1 (P = 0.09); I² = 65%
Test for overall effect: Z = 1.60 (P = 0.11)
0.7 0.85 1 1.2 1.5
Favours [experimental] Favours [control]
D
PS 0
Study or Subgroup log[Hazard Ratio] SE Weight Hazard Ratio IV, Fixed, 95% CI Hazard Ratio IV, Fixed, 95% CI
Jotte 2020 IMpower131 -0.3838 0.1477 25.5% 0.68 [0.51, 0.91]
Nishio 2020 IMpower132 -0.5746 0.1531 23.8% 0.56 [0.42, 0.76]
Socinski 2018 IMpower150 (ABCP vs BCP) -0.5964 0.1506 24.6% 0.55 [0.41, 0.74]
West 2019 IMpower130 -0.5347 0.1461 26.1% 0.59 [0.44, 0.78]
Total (95% CI) 100.0% 0.59 [0.51, 0.69]
Heterogeneity: Chi² = 1.24, df = 3 (P = 0.74); I² = 0%
Test for overall effect: Z = 6.98 (P < 0.00001)
0.5 0.7 1 1.5 2
Favours [experimental] Favours [control]
PS 1
Study or Subgroup log[Hazard Ratio] SE Weight Hazard Ratio IV, Fixed, 95% CI Hazard Ratio IV, Fixed, 95% CI
Jotte 2020 IMpower131 -0.3565 0.1049 29.0% 0.70 [0.57, 0.86]
Nishio 2020 IMpower132 -0.4679 0.1211 21.8% 0.63 [0.49, 0.79]
Socinski 2018 IMpower150 (ABCP vs BCP) -0.4506 0.1096 26.6% 0.64 [0.51, 0.79]
West 2019 IMpower130 -0.3835 0.1187 22.7% 0.68 [0.54, 0.86]
Total (95% CI) 100.0% 0.66 [0.59, 0.74]
Heterogeneity: Chi² = 0.67, df = 3 (P = 0.88); I² = 0%
Test for overall effect: Z = 7.29 (P < 0.00001)
0.5 0.7 1 1.5 2
Favours [experimental] Favours [control]
E
Active or previous smokers
Study or Subgroup log[Hazard Ratio] SE Weight Hazard Ratio IV, Fixed, 95% CI Hazard Ratio IV, Fixed, 95% CI
Jotte 2020 IMpower131 -0.357 0.0871 29.3% 0.70 [0.59, 0.83]
Nishio 2020 IMpower132 -0.4971 0.1 22.2% 0.61 [0.50, 0.74]
Socinski 2018 IMpower150 (ABCP vs BCP) -0.5453 0.0963 24.0% 0.58 [0.48, 0.70]
West 2019 IMpower130 -0.4481 0.0953 24.5% 0.64 [0.53, 0.77]
Total (95% CI) 100.0% 0.63 [0.58, 0.70]
Heterogeneity: Chi² = 2.33, df = 3 (P = 0.51); I² = 0%
Test for overall effect: Z = 9.66 (P < 0.00001)
0.5 0.7 1 1.5 2
Favours [experimental] Favours [control]
Never smoker
Study or Subgroup log[Hazard Ratio] SE Weight Hazard Ratio IV, Fixed, 95% CI Hazard Ratio IV, Fixed, 95% CI
Jotte 2020 IMpower131 -0.2585 0.3144 18.7% 0.77 [0.42, 1.43]
Nishio 2020 IMpower132 -0.7061 0.2892 22.1% 0.49 [0.28, 0.87]
Socinski 2018 IMpower150 (ABCP vs BCP) -0.2235 0.2196 38.3% 0.80 [0.52, 1.23]
West 2019 IMpower130 -0.4682 0.2967 21.0% 0.63 [0.35, 1.12]
Total (95% CI) 100.0% 0.68 [0.52, 0.89]
Heterogeneity: Chi² = 2.01, df = 3 (P = 0.57); I² = 0%
Test for overall effect: Z = 2.85 (P = 0.004)
0.5 0.7 1 1.5 2
Favours [experimental] Favours [control]
F
With liver metastases
Study or Subgroup log[Hazard Ratio] SE Weight Hazard Ratio IV, Random, 95% CI Hazard Ratio IV, Random, 95% CI
Jotte 2020 IMpower131 -0.2604 0.1815 23.2% 0.77 [0.54, 1.10]
Nishio 2020 IMpower132 -0.2659 0.2495 17.3% 0.77 [0.47, 1.25]
Reck 2019 IMpower150 (ABCP vs BCP) -0.9017 0.2194 19.7% 0.41 [0.26, 0.62]
Reck 2019 IMpower150 (ACP vs BCP) -0.2063 0.2025 21.2% 0.81 [0.55, 1.21]
West 2019 IMpower130 -0.0712 0.2329 18.6% 0.93 [0.59, 1.47]
Total (95% CI) 100.0% 0.71 [0.54, 0.94]
Heterogeneity: Tau² = 0.05; Chi² = 8.60, df = 4 (P = 0.07); I² = 54%
Test for overall effect: Z = 2.42 (P = 0.02)
0.5 0.7 1 1.5 2
Favours [experimental] Favours [control]
Without liver metastases
Study or Subgroup log[Hazard Ratio] SE Weight Hazard Ratio IV, Fixed, 95% CI Hazard Ratio IV, Fixed, 95% CI
Jotte 2020 IMpower131 -0.3891 0.0973 25.7% 0.68 [0.56, 0.82]
Nishio 2020 IMpower132 -0.5738 0.1034 22.8% 0.56 [0.46, 0.69]
Socinski 2018 IMpower150 (ABCP vs BCP) -0.4576 0.1001 24.3% 0.63 [0.52, 0.77]
West 2019 IMpower130 -0.5279 0.0946 27.2% 0.59 [0.49, 0.71]
Total (95% CI) 100.0% 0.62 [0.56, 0.68]
Heterogeneity: Chi² = 1.99, df = 3 (P = 0.57); I² = 0%
Test for overall effect: Z = 9.84 (P < 0.00001)
0.5 0.7 1 1.5 2
Favours [experimental] Favours [control]
G
EGFR mutant
Study or Subgroup log[Hazard Ratio] SE Weight Hazard Ratio IV, Random, 95% CI Hazard Ratio IV, Random, 95% CI
Reck 2019 IMpower150 (ABCP vs BCP) -0.496 0.2682 47.4% 0.61 [0.36, 1.03]
Reck 2019 IMpower150 (ACP vs BCP) 0.131 0.2274 52.6% 1.14 [0.73, 1.78]
Total (95% CI) 100.0% 0.85 [0.46, 1.56]
Heterogeneity: Tau² = 0.13; Chi² = 3.18, df = 1 (P = 0.07); I² = 69%
Test for overall effect: Z = 0.53 (P = 0.60)
0.5 0.7 1 1.5 2
Favours [experimental] Favours [control]
EGFR wildtype
Study or Subgroup log[Hazard Ratio] SE Weight Hazard Ratio IV, Fixed, 95% CI Hazard Ratio IV, Fixed, 95% CI
Socinski 2018 IMpower150 (ABCP vs BCP) -0.4775 0.09 100.0% 0.62 [0.52, 0.74]
Total (95% CI) 100.0% 0.62 [0.52, 0.74]
Heterogeneity: Not applicable
Test for overall effect: Z = 5.31 (P < 0.00001)
0.5 0.7 1 1.5 2
Favours [experimental] Favours [control]

H

Asian

Study or Subgroup	log[Hazard Ratio]	SE	Weight	Hazard Ratio IV, Random, 95% CI
Jotte 2020 IMpower131	-0.3675	0.2588	45.2%	0.69 [0.42, 1.15]
Nishio 2020 IMpower132	-0.8675	0.2069	54.8%	0.42 [0.28, 0.63]
**Total (95% CI)**			**100.0%**	**0.53 [0.32, 0.86]**

Heterogeneity: Tau² = 0.07; Chi² = 2.28, df = 1 (P = 0.13); I² = 56%
Test for overall effect: Z = 2.58 (P = 0.010)

Hazard Ratio IV, Random, 95% CI: 0.5 0.7 1 1.5 2
Favours [experimental] Favours [control]

Black or African American

Study or Subgroup	log[Hazard Ratio]	SE	Weight	Hazard Ratio IV, Fixed, 95% CI
Jotte 2020 IMpower131	-0.9347	0.8515	100.0%	0.39 [0.07, 2.08]
**Total (95% CI)**			**100.0%**	**0.39 [0.07, 2.08]**

Heterogeneity: Not applicable
Test for overall effect: Z = 1.10 (P = 0.27)

Hazard Ratio IV, Fixed, 95% CI: 0.1 0.2 0.5 1 2 5 10
Favours [experimental] Favours [control]

White

Study or Subgroup	log[Hazard Ratio]	SE	Weight	Hazard Ratio IV, Fixed, 95% CI
Jotte 2020 IMpower131	-0.351	0.0901	61.0%	0.70 [0.59, 0.84]
Nishio 2020 IMpower132	-0.3953	0.1127	39.0%	0.67 [0.54, 0.84]
**Total (95% CI)**			**100.0%**	**0.69 [0.60, 0.79]**

Heterogeneity: Chi² = 0.09, df = 1 (P = 0.76); I² = 0%
Test for overall effect: Z = 5.23 (P < 0.00001)

Hazard Ratio IV, Fixed, 95% CI: 0.7 0.85 1 1.2 1.5
Favours [experimental] Favours [control]

I

PD-L1 expression on <1% of TC and IC (TC0 and IC0)

Study or Subgroup	log[Hazard Ratio]	SE	Weight	Hazard Ratio IV, Random, 95% CI
Fehrenbacher 2016 POPLAR	0.1173	0.2303	12.4%	1.12 [0.72, 1.77]
Fehrenbacher 2018 OAK	0.1079	0.0943	19.4%	1.11 [0.93, 1.34]
Jotte 2020 IMpower131	-0.2084	0.1214	18.0%	0.81 [0.64, 1.03]
Nishio 2020 IMpower132	-0.8087	0.1849	14.6%	0.45 [0.31, 0.64]
Socinski 2018 IMpower150 (ABCP vs BCP)	-0.2567	0.1238	17.9%	0.77 [0.61, 0.99]
West 2019 IMpower130	-0.3349	0.125	17.8%	0.72 [0.56, 0.91]
**Total (95% CI)**			**100.0%**	**0.80 [0.63, 1.01]**

Heterogeneity: Tau² = 0.07; Chi² = 24.50, df = 5 (P = 0.0002); I² = 80%
Test for overall effect: Z = 1.84 (P = 0.07)

Hazard Ratio IV, Random, 95% CI: 0.5 0.7 1 1.5 2
Favours [experimental] Favours [control]

PD-L1 expression on ≥1% of TC or IC (TC1/2/3 or IC1/2/3)

Study or Subgroup	log[Hazard Ratio]	SE	Weight	Hazard Ratio IV, Random, 95% CI
Fehrenbacher 2016 POPLAR	-0.1568	0.1557	16.5%	0.85 [0.63, 1.16]
Fehrenbacher 2018 OAK	-0.1428	0.0849	22.8%	0.87 [0.73, 1.02]
Jassem 2021 IMpower110	-0.3308	0.0918	22.2%	0.72 [0.60, 0.86]
Jotte 2020 IMpower131	-0.4977	0.1206	19.6%	0.61 [0.48, 0.77]
Socinski 2018 IMpower150 (ABCP vs BCP)	-0.6939	0.1264	19.0%	0.50 [0.39, 0.64]
**Total (95% CI)**			**100.0%**	**0.70 [0.57, 0.85]**

Heterogeneity: Tau² = 0.04; Chi² = 16.32, df = 4 (P = 0.003); I² = 75%
Test for overall effect: Z = 3.58 (P = 0.0003)

Hazard Ratio IV, Random, 95% CI: 0.5 0.7 1 1.5 2
Favours [experimental] Favours [control]

PD-L1 expression on ≥1% of TC or IC and <50% of TC and <10% of IC (TC1/2 or IC1/2)

Study or Subgroup	log[Hazard Ratio]	SE	Weight	Hazard Ratio IV, Fixed, 95% CI
Jotte 2020 IMpower131	-0.3591	0.1407	33.3%	0.70 [0.53, 0.92]
Nishio 2020 IMpower132	-0.2184	0.1871	18.8%	0.80 [0.56, 1.16]
Socinski 2018 IMpower150 (ABCP vs BCP)	-0.5765	0.1608	25.5%	0.56 [0.41, 0.77]
West 2019 IMpower130	-0.4986	0.1715	22.4%	0.61 [0.43, 0.85]
**Total (95% CI)**			**100.0%**	**0.66 [0.56, 0.77]**

Heterogeneity: Chi² = 2.51, df = 3 (P = 0.47); I² = 0%
Test for overall effect: Z = 5.16 (P < 0.00001)

Hazard Ratio IV, Fixed, 95% CI: 0.5 0.7 1 1.5 2
Favours [experimental] Favours [control]

PD-L1 expression on ≥5% of TC or IC (TC2/3 or IC2/3)

Study or Subgroup	log[Hazard Ratio]	SE	Weight	Hazard Ratio IV, Fixed, 95% CI
Fehrenbacher 2016 POPLAR	-0.3299	0.2169	10.9%	0.72 [0.47, 1.10]
Fehrenbacher 2018 OAK	-0.3193	0.1203	35.3%	0.73 [0.57, 0.92]
Jassem 2021 IMpower110	-0.4458	0.1262	32.1%	0.64 [0.50, 0.82]
Jotte 2020 IMpower131	-0.6155	0.1535	21.7%	0.54 [0.40, 0.73]
**Total (95% CI)**			**100.0%**	**0.65 [0.57, 0.75]**

Heterogeneity: Chi² = 2.53, df = 3 (P = 0.47); I² = 0%
Test for overall effect: Z = 5.95 (P < 0.00001)

Hazard Ratio IV, Fixed, 95% CI: 0.5 0.7 1 1.5 2
Favours [experimental] Favours [control]

PD-L1 expression on ≥50% of TC or ≥10% of IC (TC3 or IC3)

Study or Subgroup	log[Hazard Ratio]	SE	Weight	Hazard Ratio IV, Fixed, 95% CI
Fehrenbacher 2016 POPLAR	-0.5114	0.3366	6.1%	0.60 [0.31, 1.16]
Fehrenbacher 2018 OAK	-0.533	0.183	20.8%	0.59 [0.41, 0.84]
Jassem 2021 IMpower110	-0.5273	0.1615	26.7%	0.59 [0.43, 0.81]
Jotte 2020 IMpower131	-0.8259	0.2466	11.5%	0.44 [0.27, 0.71]
Nishio 2020 IMpower132	-0.7775	0.3759	4.9%	0.46 [0.22, 0.96]
Socinski 2018 IMpower150 (ABCP vs BCP)	-0.9486	0.2233	14.0%	0.39 [0.25, 0.60]
West 2019 IMpower130	-0.6701	0.2085	16.0%	0.51 [0.34, 0.77]
**Total (95% CI)**			**100.0%**	**0.52 [0.44, 0.61]**

Heterogeneity: Chi² = 3.57, df = 6 (P = 0.73); I² = 0%
Test for overall effect: Z = 7.86 (P < 0.00001)

Hazard Ratio IV, Fixed, 95% CI: 0.5 0.7 1 1.5 2
Favours [experimental] Favours [control]

J

1st line therapy

Study or Subgroup	log[Hazard Ratio]	SE	Weight	Hazard Ratio IV, Fixed, 95% CI
Jassem 2021 IMpower110	-0.3308	0.0918	19.0%	0.72 [0.60, 0.86]
Jotte 2020 IMpower131	-0.3367	0.0889	20.3%	0.71 [0.60, 0.85]
Nishio 2020 IMpower132	-0.5169	0.0961	17.4%	0.60 [0.49, 0.72]
Reck 2019 IMpower150 (ABCP vs BCP)	-0.5321	0.0822	23.7%	0.59 [0.50, 0.69]
West 2019 IMpower130	-0.4388	0.0905	19.6%	0.64 [0.54, 0.77]
**Total (95% CI)**			**100.0%**	**0.65 [0.60, 0.70]**

Heterogeneity: Chi² = 4.63, df = 4 (P = 0.33); I² = 14%
Test for overall effect: Z = 10.82 (P < 0.00001)

Hazard Ratio IV, Fixed, 95% CI: 0.5 0.7 1 1.5 2
Favours [experimental] Favours [control]

≥2nd line therapy

Study or Subgroup	log[Hazard Ratio]	SE	Weight	Hazard Ratio IV, Fixed, 95% CI
Fehrenbacher 2016 POPLAR	-0.0607	0.1366	16.7%	0.94 [0.72, 1.23]
Fehrenbacher 2018 OAK	-0.0428	0.0611	83.3%	0.96 [0.85, 1.08]
**Total (95% CI)**			**100.0%**	**0.96 [0.86, 1.07]**

Heterogeneity: Chi² = 0.01, df = 1 (P = 0.90); I² = 0%
Test for overall effect: Z = 0.82 (P = 0.41)

Hazard Ratio IV, Fixed, 95% CI: 0.85 0.9 1 1.1 1.2
Favours [experimental] Favours [control]

Figure S2: Forest plots of HRs comparing PFS between atezolizumab-based therapy and chemotherapy-based therapy with respect to (A) age group, (B) gender, (C) histological type, (D) PS score, (E) smoking status, (F) liver metastases status, (G) EGFR mutationstatus, (H) race, (I) PD-L1 expression, and (J) treatment line.

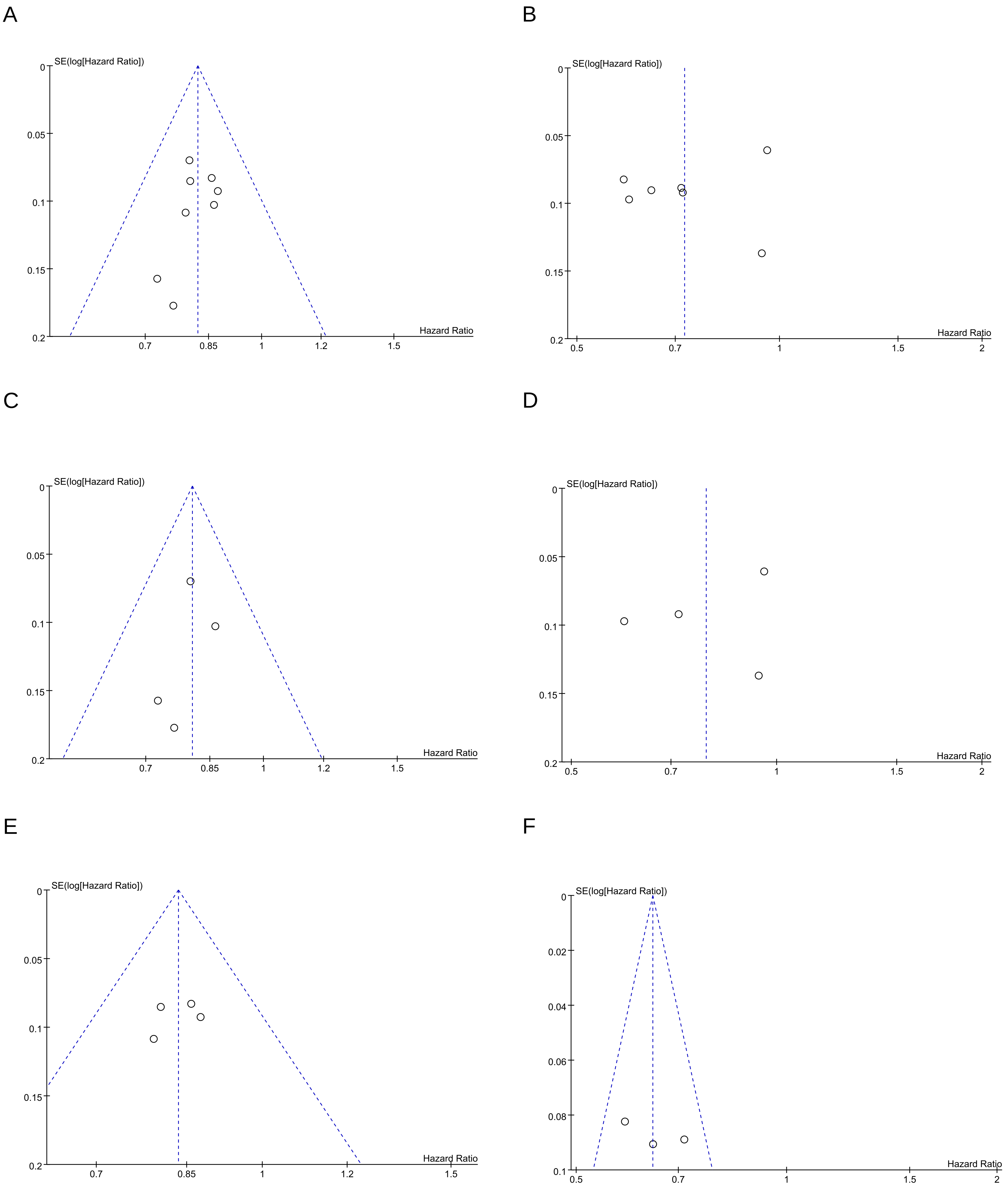

Figure S3: Funnel plots for (A) OS and (B) PFS between atezolizumab-based therapy and chemotherapy-based therapy, (C) OS and (D) PFS based on atezolizumab monotherapy, (E) OS and (F) PFS based on atezolizumab combined therapy.

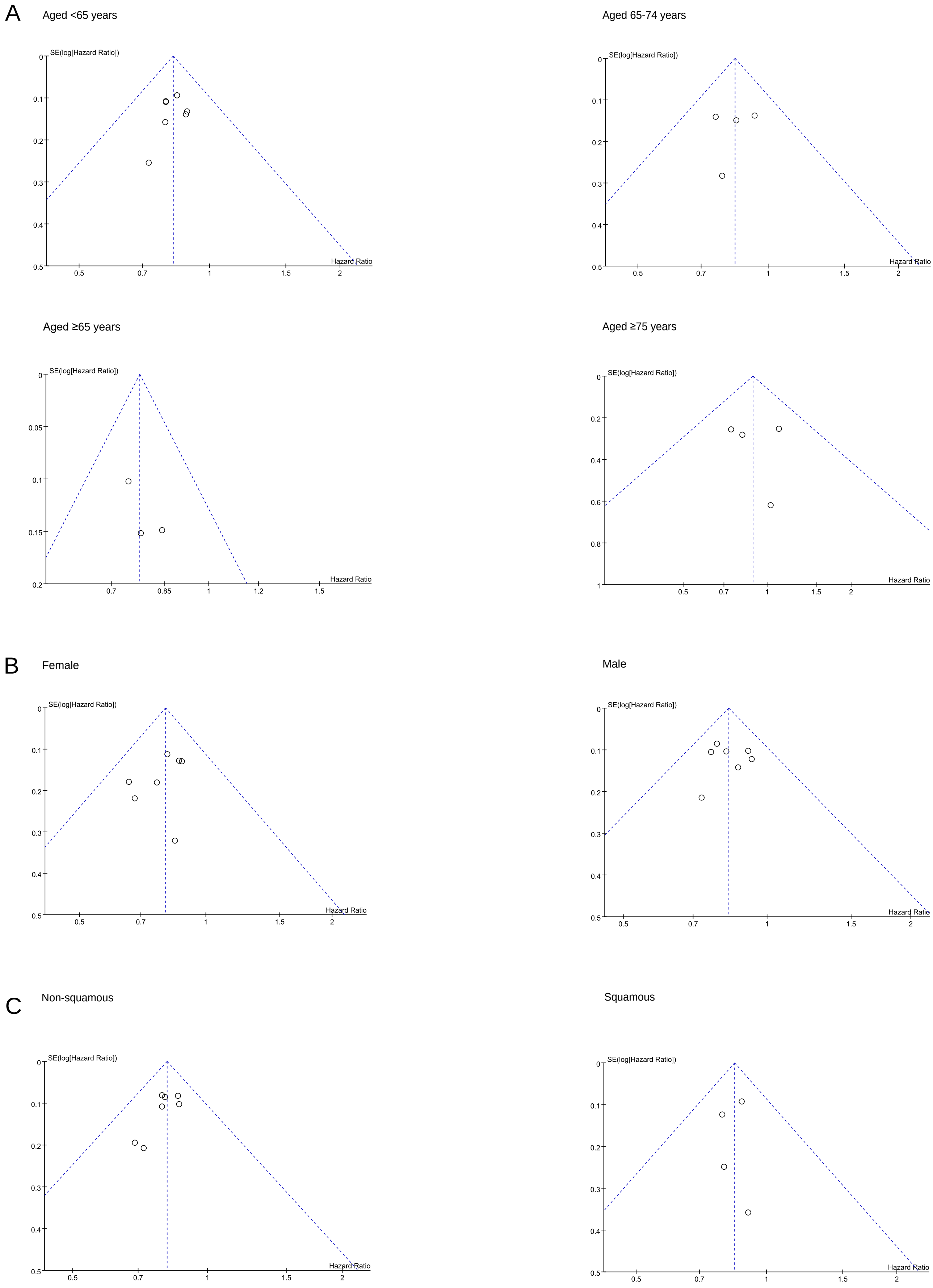

A
Aged <65 years
Aged 65-74 years
Aged ≥65 years
Aged ≥75 years
B
Female
Male
C
Non-squamous
Squamous
SE(log[Hazard Ratio])
Hazard Ratio

D

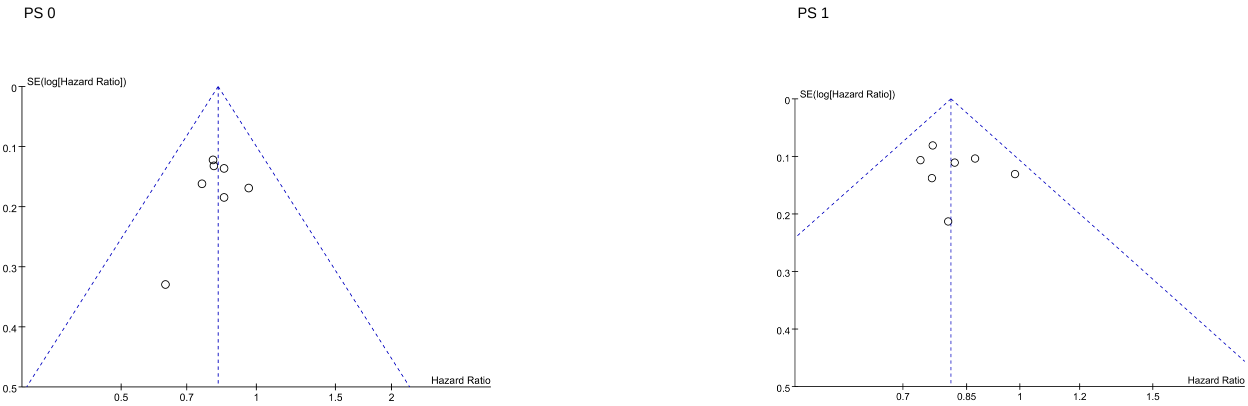
PS 0
SE(log[Hazard Ratio])
0
0.1
0.2
0.3
0.4
0.5
Hazard Ratio
0.5
0.7
1
1.5
2
PS 1
SE(log[Hazard Ratio])
0
0.1
0.2
0.3
0.4
0.5
Hazard Ratio
0.7
0.85
1
1.2
1.5

E

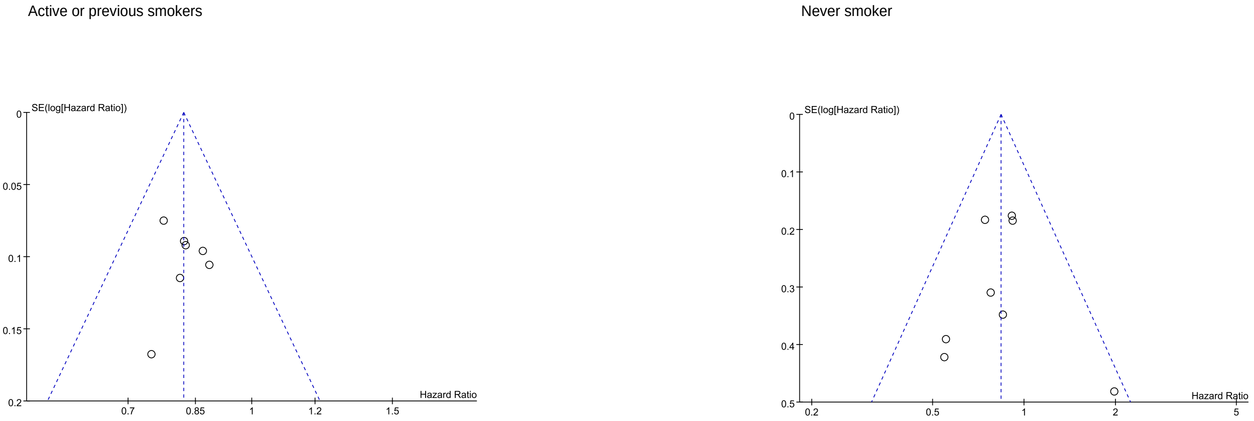
Active or previous smokers
SE(log[Hazard Ratio])
0
0.05
0.1
0.15
0.2
Hazard Ratio
0.7
0.85
1
1.2
1.5
Never smoker
SE(log[Hazard Ratio])
0
0.1
0.2
0.3
0.4
0.5
Hazard Ratio
0.2
0.5
1
2
5

F

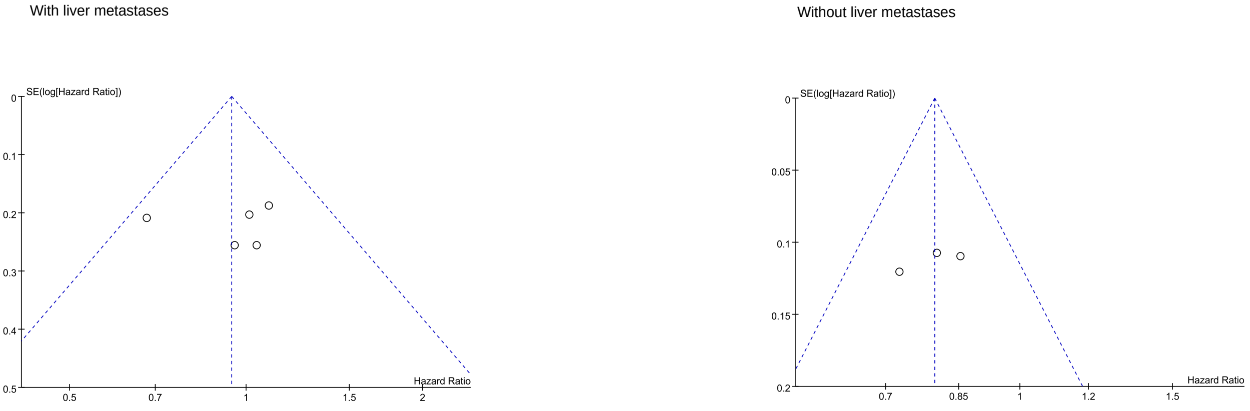
With liver metastases
SE(log[Hazard Ratio])
0
0.1
0.2
0.3
0.4
0.5
Hazard Ratio
0.5
0.7
1
1.5
2
Without liver metastases
SE(log[Hazard Ratio])
0
0.05
0.1
0.15
0.2
Hazard Ratio
0.7
0.85
1
1.2
1.5

G

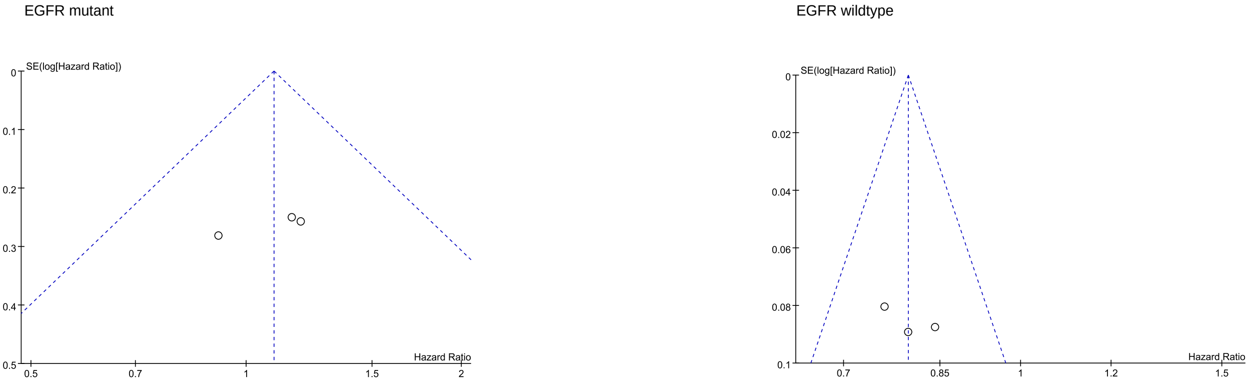
EGFR mutant
SE(log[Hazard Ratio])
0
0.1
0.2
0.3
0.4
0.5
Hazard Ratio
0.5
0.7
1
1.5
2
EGFR wildtype
SE(log[Hazard Ratio])
0
0.02
0.04
0.06
0.08
0.1
Hazard Ratio
0.7
0.85
1
1.2
1.5

H

Asian

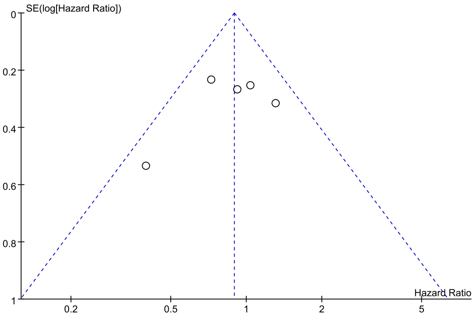

Black or African American

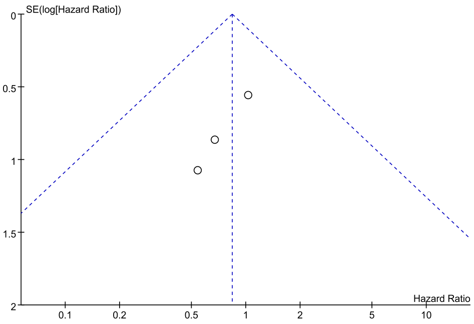

White

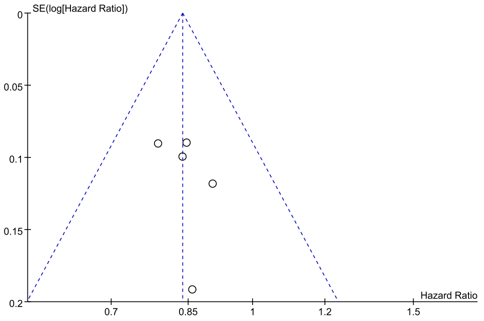

I

PD-L1 expression on <1% of TC and IC (TC0 and IC0)

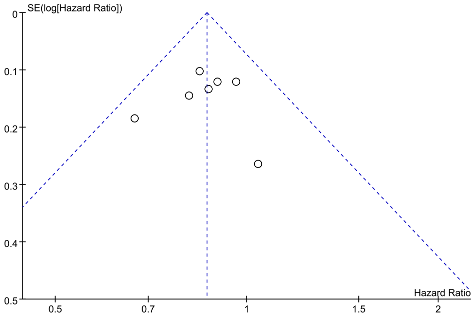

PD-L1 expression on ≥1% of TC or IC (TC1/2/3 or IC1/2/3)

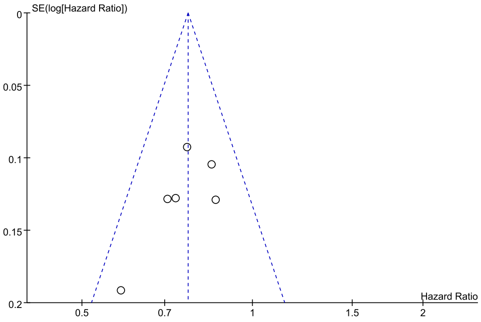

PD-L1 expression on ≥1% of TC or IC and <50% of TC and <10% of IC (TC1/2 or IC1/2)

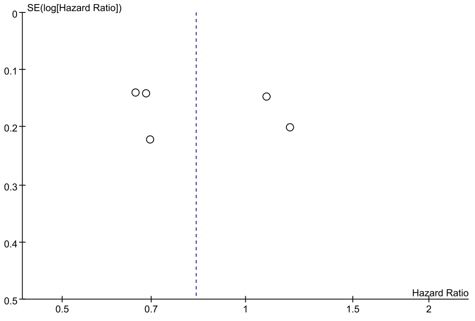

PD-L1 expression on ≥5% of TC or IC (TC2/3 or IC2/3)

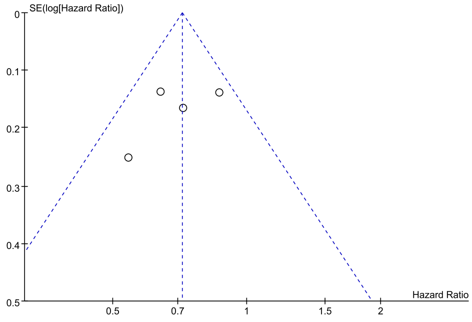

PD-L1 expression on ≥50% of TC or ≥10% of IC (TC3 or IC3)

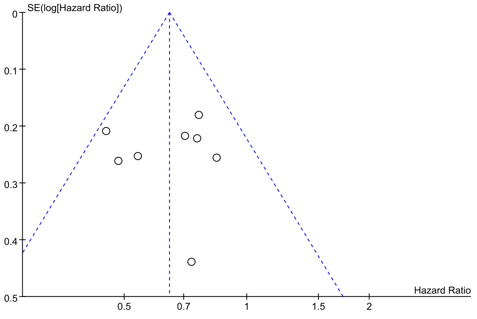

J

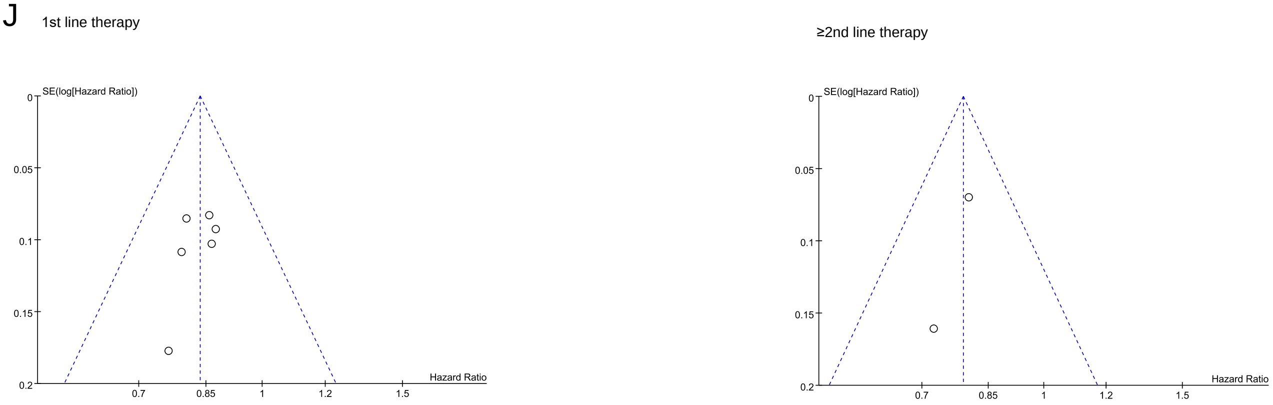

Figure S4: Funnel plots for OS in the subgroup with respect to (A) age group, (B) gender, (C) histological type, (D) PS score, (E) smoking status, (F) liver metastases status, (G)EGFR mutation status, (H) race, (I) PD-L1expression, and (J) treatment line.

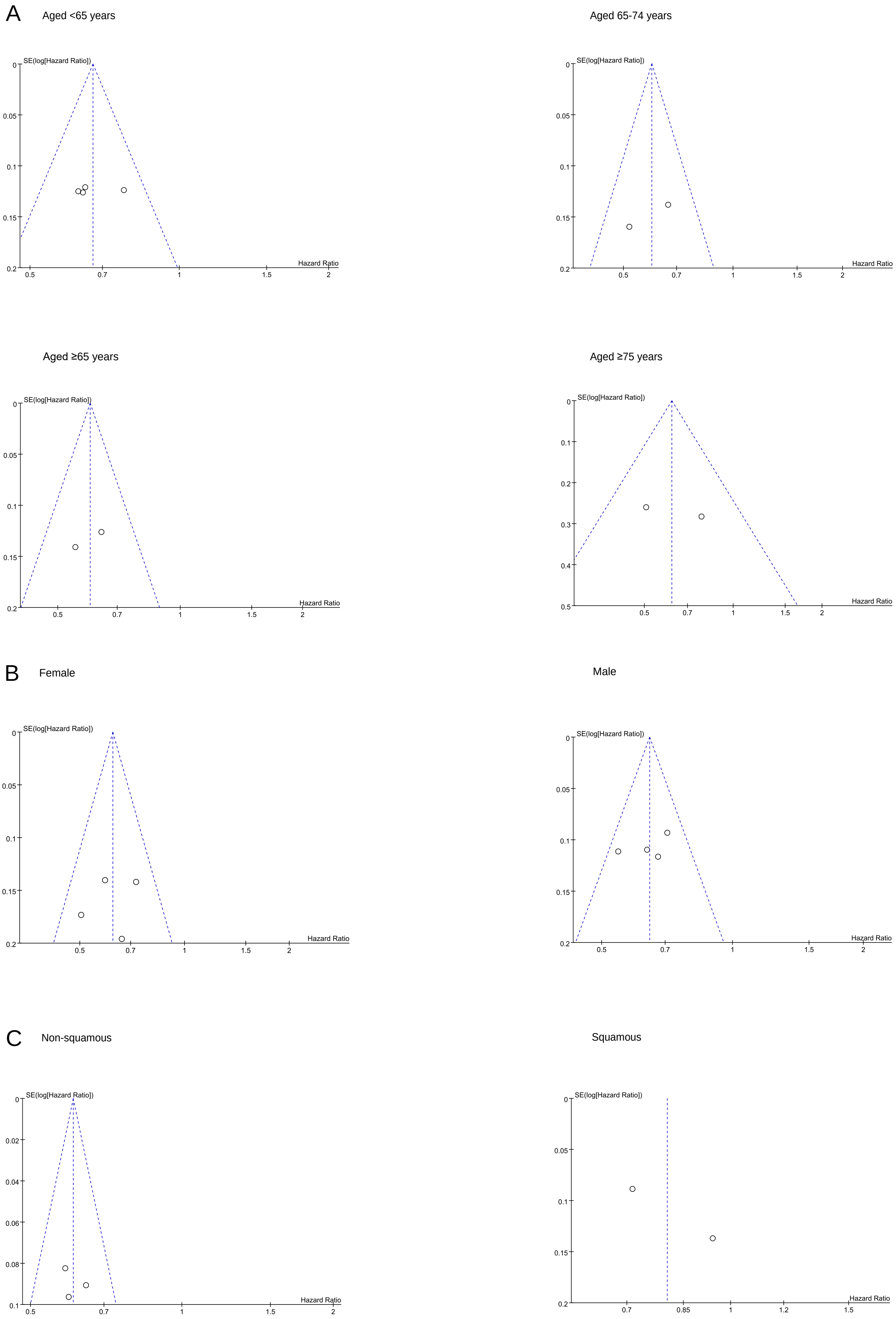
A
Aged <65 years
SE(log[Hazard Ratio])
0
0.05
0.1
0.15
0.2
Hazard Ratio
0.5
0.7
1
1.5
2
Aged 65-74 years
SE(log[Hazard Ratio])
Hazard Ratio
Aged ≥65 years
SE(log[Hazard Ratio])
Hazard Ratio
Aged ≥75 years
SE(log[Hazard Ratio])
0
0.1
0.2
0.3
0.4
0.5
Hazard Ratio
B
Female
SE(log[Hazard Ratio])
Hazard Ratio
Male
SE(log[Hazard Ratio])
Hazard Ratio
C
Non-squamous
SE(log[Hazard Ratio])
0
0.02
0.04
0.06
0.08
0.1
Hazard Ratio
Squamous
SE(log[Hazard Ratio])
Hazard Ratio
0.7
0.85
1
1.2
1.5

D PS 0 PS 1

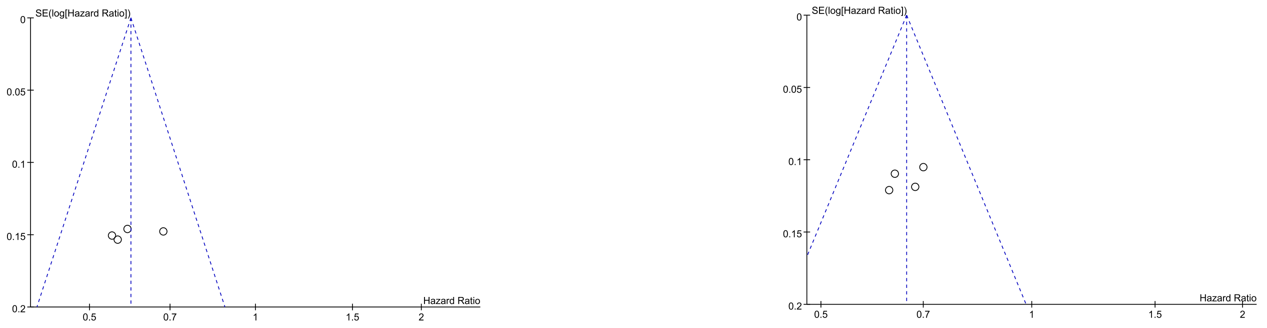

E Active or previous smokers Never smoker

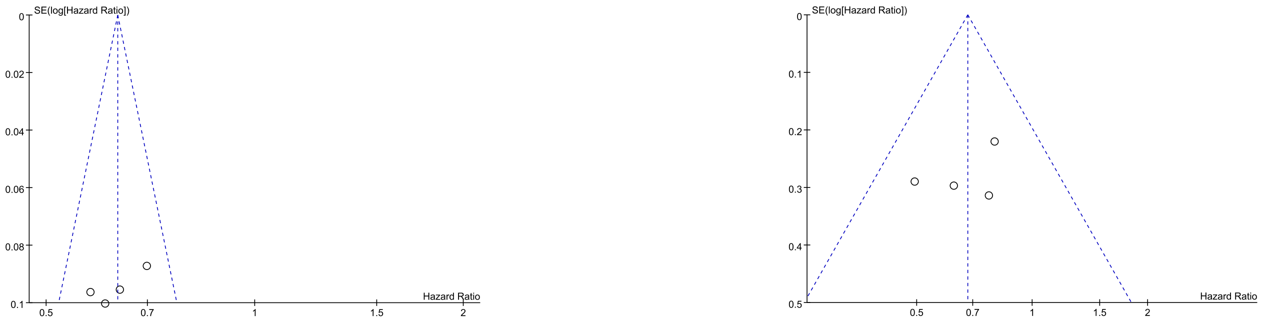

F With liver metastases Without liver metastases

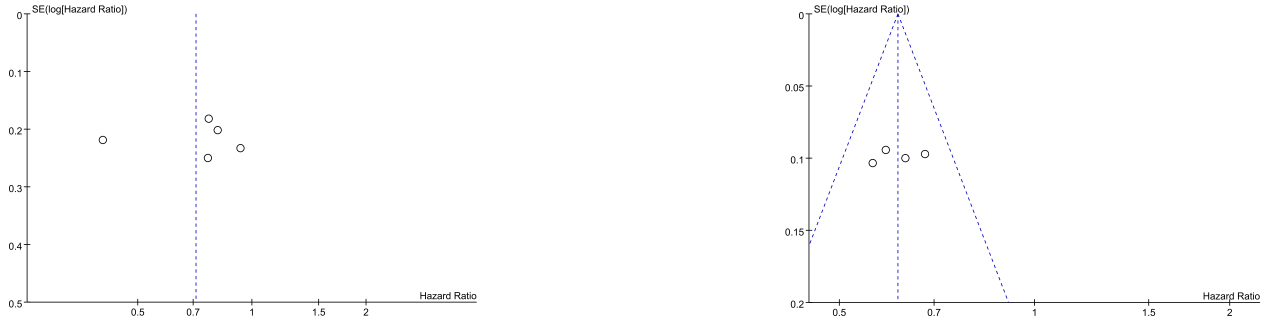

G EGFR mutant EGFR wildtype

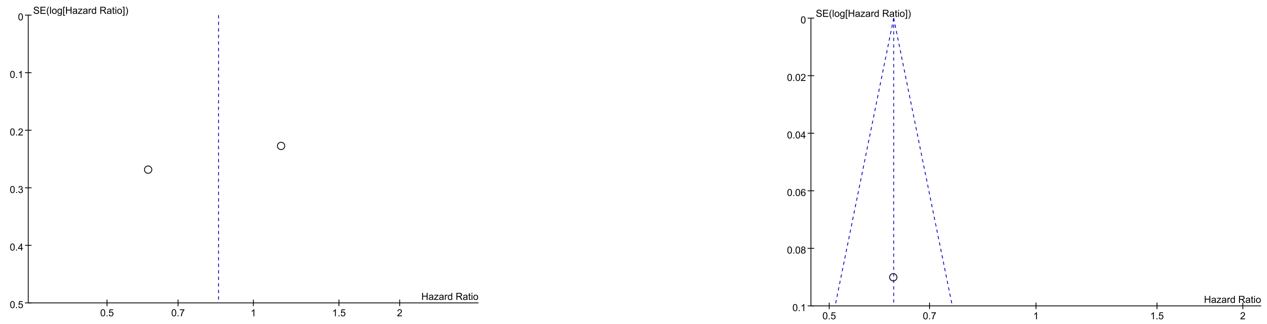

H

Asian

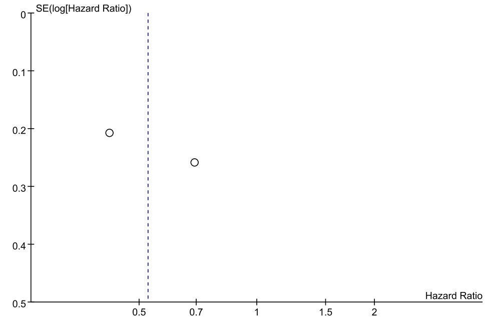

Black or African American

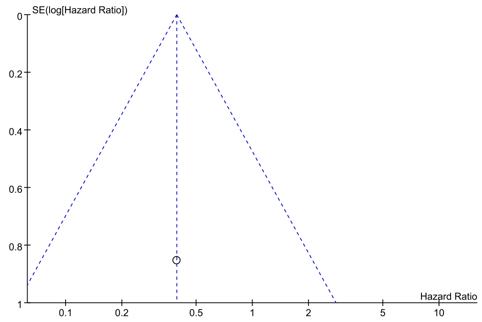

White

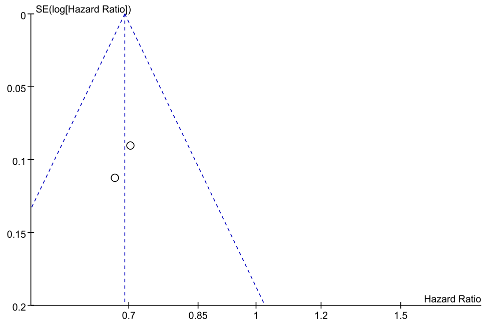

I

PD-L1 expression on <1% of TC and IC (TC0 and IC0)

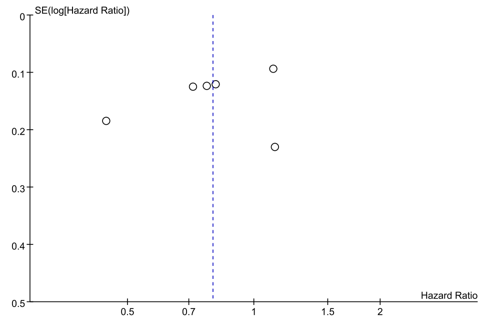

PD-L1 expression on ≥1% of TC or IC (TC1/2/3 or IC1/2/3)

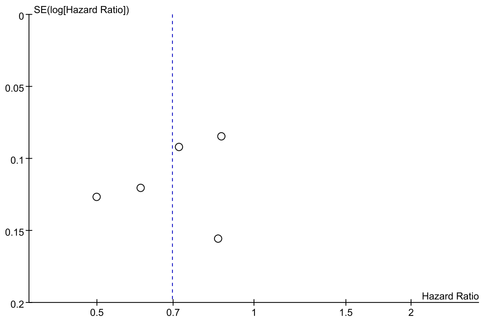

PD-L1 expression on ≥1% of TC or IC and <50% of TC and <10% of IC (TC1/2 or IC1/2)

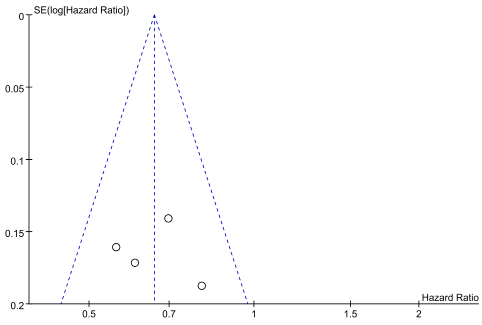

PD-L1 expression on ≥5% of TC or IC (TC2/3 or IC2/3)

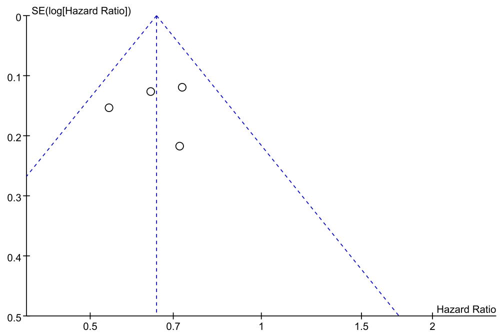

PD-L1 expression on ≥50% of TC or ≥10% of IC (TC3 or IC3)

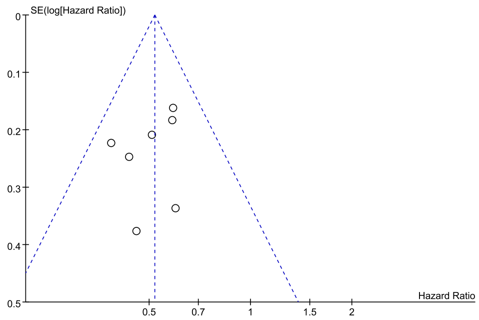

J 1st line therapy

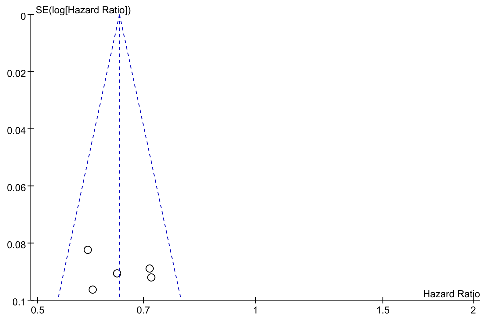

≥2nd line therapy

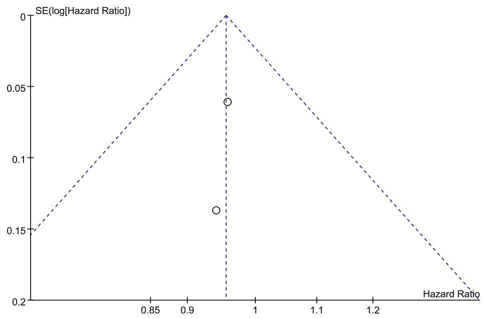

Figure S5: Funnel plots for PFS in the subgroup with respect to (A) age group, (B) gender, (C) histological type, (D) PS score, (E) smoking status, (F) liver metastases status, (G)EGFR mutation status, (H) race, (I) PD-L1 expression, and (J) treatment line.
